# Antibodies exhibit multiple paratope states influencing V_H_–V_L_ domain orientations

**DOI:** 10.1038/s42003-020-01319-z

**Published:** 2020-10-20

**Authors:** Monica L. Fernández-Quintero, Nancy D. Pomarici, Barbara A. Math, Katharina B. Kroell, Franz Waibl, Alexander Bujotzek, Guy Georges, Klaus R. Liedl

**Affiliations:** 1 Institute of General, Inorganic and Theoretical Chemistry, and Center for Molecular Biosciences Innsbruck (CMBI), University of Innsbruck, Innrain 80-82, A-6020 Innsbruck, Austria; 2 Roche Pharma Research and Early Development, Large Molecule Research, Roche Innovation Center Munich, Penzberg, Germany

**Keywords:** Biophysics, Computational biophysics, Computational biology and bioinformatics, Computational models, Protein structure predictions

## Abstract

In the last decades, antibodies have emerged as one of the most important and successful classes of biopharmaceuticals. The highest variability and diversity of an antibody is concentrated on six hypervariable loops, also known as complementarity determining regions (CDRs) shaping the antigen-binding site, the paratope. Whereas it was assumed that certain sequences can only adopt a limited set of backbone conformations, in this study we present a kinetic classification of several paratope states in solution. Using molecular dynamics simulations in combination with experimental structural information we capture the involved conformational transitions between different canonical clusters and additional dominant solution structures occurring in the micro-to-millisecond timescale. Furthermore, we observe a strong correlation of CDR loop movements. Another important aspect when characterizing different paratope states is the relative V_H_/V_L_ orientation and the influence of the distinct CDR loop states on the V_H_/V_L_ interface. Conformational rearrangements of the CDR loops do not only have an effect on the relative V_H_/V_L_ orientations, but also influence in some cases the elbow-angle dynamics and shift the respective distributions. Thus, our results show that antibodies exist as several interconverting paratope states, each contributing to the antibody’s properties.

## Abstract (web summary)

Fernández-Quintero et al. employ molecular dynamics simulations in combination with experimental structural information to demonstrate that antibodies exist as several interconverting paratope states. They propose that dynamic conformational transitions on the micro-to-millisecond timescale are responsible for antibody allostery in contrast to the long believed paradigm of static canonical structures determining binding properties and specificity of antibodies.

## Introduction

Antibodies have become one of the fastest-growing class of biopharmaceuticals and are key players as therapeutic agents because of their ability to bind a variety of targets^1–4^. Therefore, the importance to characterize and engineer the structure of antibodies to improve specificity, stability, and suitability as biotherapeutics increased substantially. The antibody binding site consists of six hypervariable loops, each three on the variable domains of the heavy (V_H_) and the light chain (V_L_), that shape the antigen-binding site, the paratope^5–7^. Five of the six CDR loops, except for the CDR-H3 loop, have been classified into canonical clusters, assuming that depending on the length and sequence, antibody CDR loops can only adopt a limited number of backbone conformations^8–11^. The highest diversity in length, sequence, and structure can be observed for the CDR-H3 loop^9,12^. Due to its ability to adopt various distinct conformations during the V(D)J recombination and somatic hyper-mutation, the CDR-H3 loop structure prediction still remains challenging^13–19^. Together with the CDR-H3 loop, the CDR-L3 loop is located in the center of the paratope and contributes to antigen recognition. The diversity of the CDR-L3 loop is comparable to the CDR-H3 loop, however without the contribution of a D gene, the variability is lower^20,21^. With the exponential rise of antibody structures the number of antibody databases and sequence-based classification servers increased substantially^1,22^. Numerous studies tried to classify antibody CDR loops based on their sequence and structure and to correlate them with their locus to improve fast antibody structure prediction and design^22–26^. Recent studies using molecular dynamics simulations extended the model of static canonical clusters to a characterization of the CDR-L3 and CDR-H3 loops as ensembles in solution by showing that for a given CDR-L3/CDR-H3 loop sequence several conformations should be considered. Besides capturing several conformational transitions between different canonical clusters, other probable CDR-L3 loop structures in solution were identified that are not apparent from X-ray analysis most likely due to crystal packing effects^27,28^. Another crucial aspect in antibody structure design is the relative orientation of the two variable domains V_H_/V_L_, which influences the shape of the paratope and plays an important role in understanding antigen-specificity^29,30^. The variability in the relative orientation of the V_H_ and V_L_ domains is an additional structural feature of antibodies, which increases the effective size of the antibody repertoire. This is in line with the concept of conformational diversity that the same antibody can adopt various conformations, which influences the binding properties^31–33^. To characterize and understand favorable orientations of the V_H_ and V_L_ domains, where essential solution structure recognizing different antigens, the considerable binding site flexibility in the low nanosecond timescale should be considered^30^. It has been shown that conformational rearrangements of the binding site might not only have an effect on the relative V_H_ and V_L_ orientations but also on the elbow-angle^34,35^.

In this study, we analyze the conformational diversity of all CDR loops to identify transition probabilities between distinct paratope rearrangements and kinetically characterize the CDR loop ensembles in solution. Additionally, we investigate correlated movements between different CDR loops and show the influence of distinct paratope states on the relative V_H_ and V_L_ orientations and on the elbow-angle dynamics. The set of antibodies investigated in this study are chosen based on strong experimental information (the availability of highly resolved crystal structures, affinity, specificity). Additionally, we preferred an assignment to different canonical clusters, to investigate the effect of affinity maturation, multispecificity, different light chain types on the CDR loop ensemble, the relative interdomain orientations, and the elbow-angle dynamics.

## Results

### Anti-polyhydroxybutyrate antibody

**Fig. 1:**
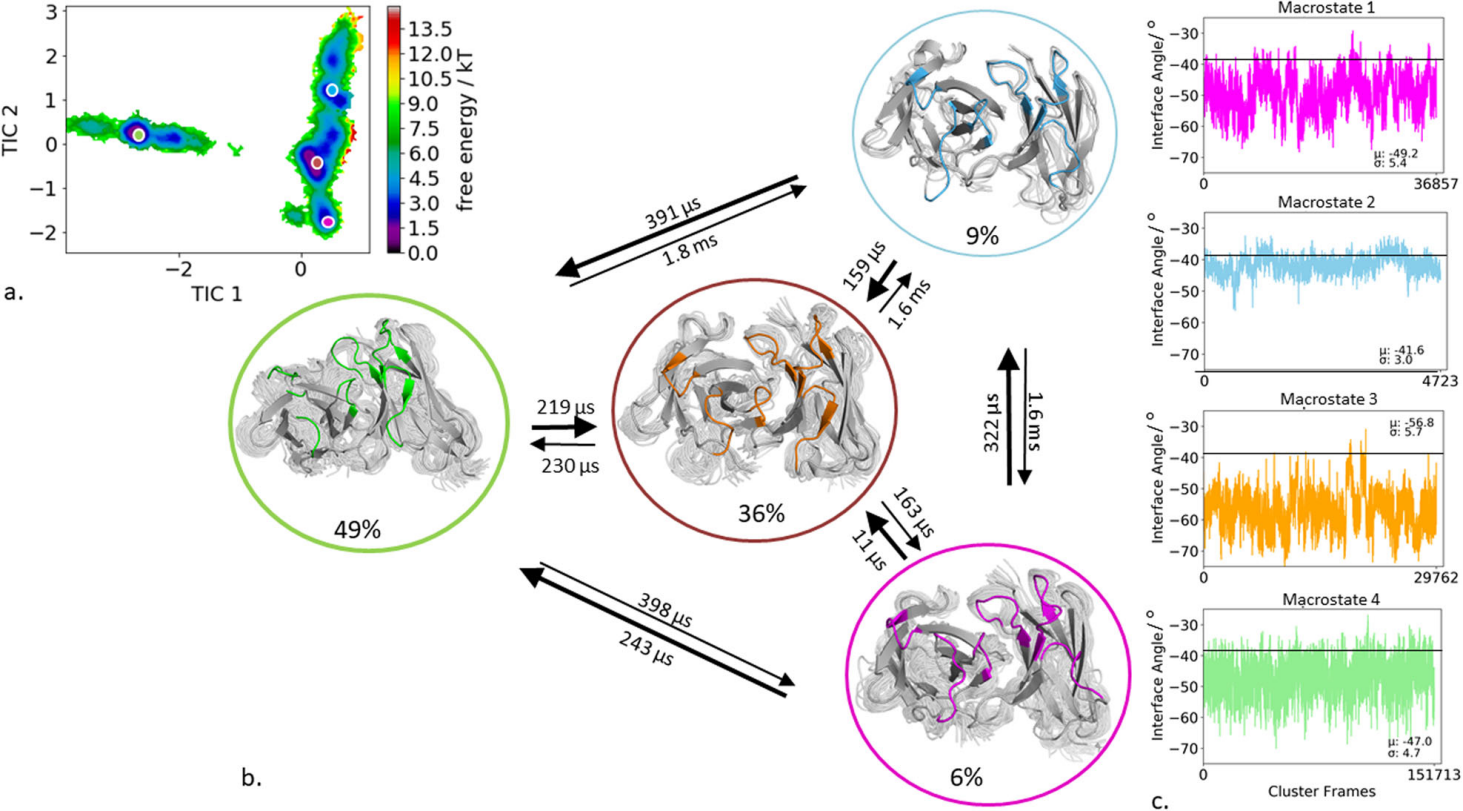
Paratope states of the anti-polyhydroxybutyrate antibody Fv including the results of the Markov-state model and shows the relative V_H_–V_L_ interface distributions for the respective macrostates. **a** Combined free energy landscape of all CDR loops with the respective four macrostate representatives. **b** Markov-state model, transition kinetics, and state probabilities of different paratope states for the anti-polyhydroxybutyrate antibody. **c** Relative V_H_–V_L_ interface dynamics of the macrostate ensembles, color-coded according to the Markov-state model in (**b**). The horizontal line depicts the interface orientation of the crystal structure.

The first antibody studied is the human anti-polyhydroxybutyrate antibody variable fragment (PDB accession code: 2D7T), which binds with high affinity to the biodegradable polymer film. As described in the “methods” section we clustered the obtained 200 ns bias-exchange metadynamics simulation and used the resulting 254 cluster representatives as starting structures for each 100 ns molecular dynamics simulations. This approach does not only allow us to generate a broad ensemble of CDR loop conformations but also to characterize the paratope states in solution kinetically. Figure 1a shows the resulting tICA free energy surface of the 25.4 µs of trajectories of all CDR loops combined to identify kinetically relevant paratope states. The state probabilities, transition kinetics, and the respective macrostate ensembles are shown in Fig. 1b. We obtained four macrostates with CDR binding loop transition timescales up to the millisecond timescale. Additionally, these four macrostate ensembles were then used to calculate the respective interface angle distributions to investigate if CDR loop rearrangements change the relative V_H_ and V_L_ orientation (Fig. 1c). We clearly see that different paratope loop states shift the relative interdomain orientation distributions. The combined free energy surface of all CDR loops reveals a strong macrostate separation in the tIC1, which describes conformational changes of the CDR-H3, the CDR-H1, the CDR-H2, and CDR-L2 loops. Additionally, all four macrostates are further separated by the tIC2 which describes conformational transitions in the CDR-H3, CDR-H1, and CDR-L2 loops.

**Fig. 2:**
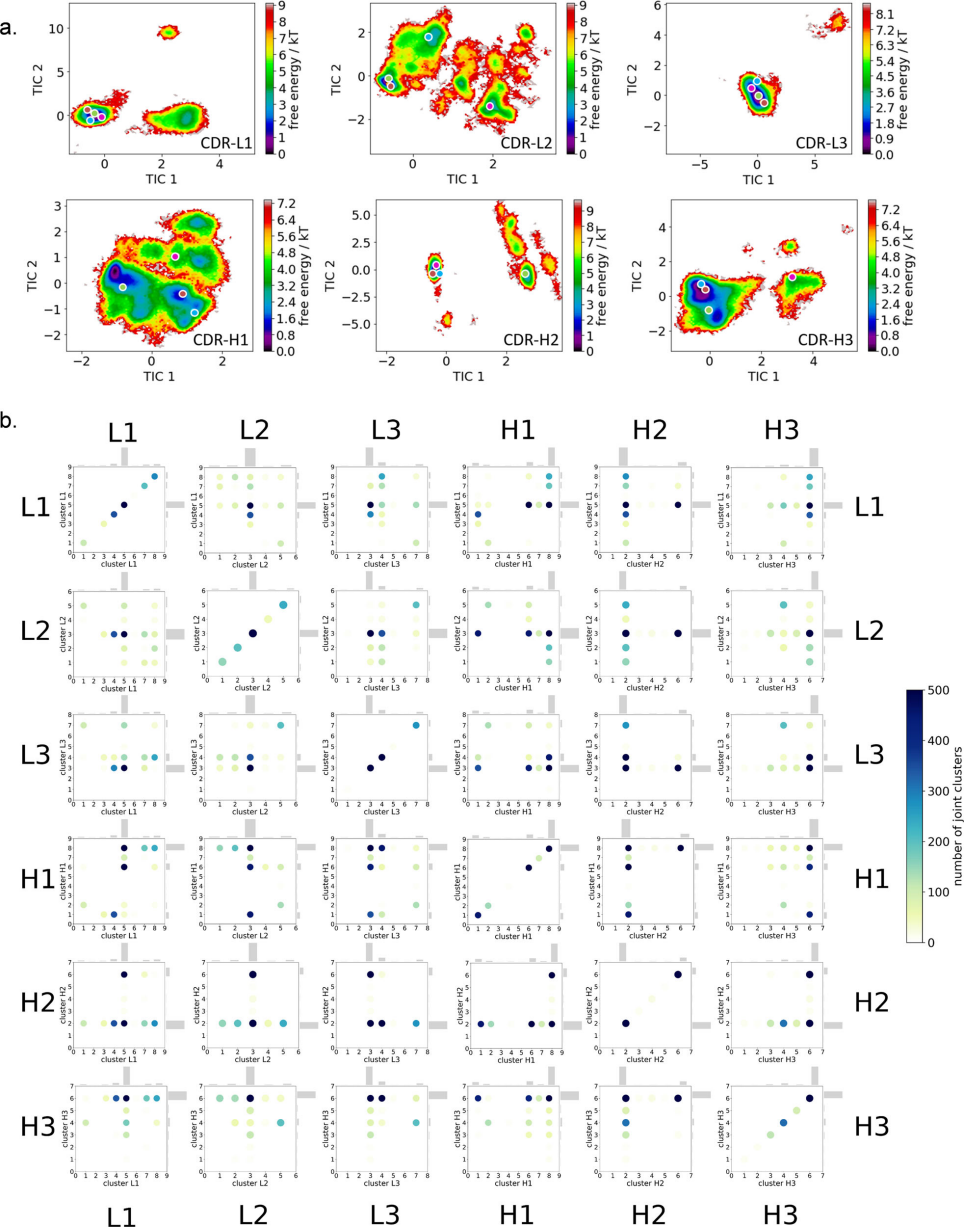
Free energy surfaces and loop correlations for all individual CDR loops, showing which loops contribute to conformational rearrangements in the different paratope states. **a** Free energy surfaces of all individual CDR loops of the anti-polyhydroxybutyrate antibody including the projections of macrostates of Fig. 1b). **b** Visualization of simultaneous occurrence of CDR loop conformations for all CDR loops.

The free energy landscapes of the individual CDR loops are shown in Fig. 2a, including the projections of the paratope macrostate representatives of Fig. 1b to investigate correlated loop movements and to identify the involved loop rearrangements occurring within the four paratope states.

Besides classifying the states kinetically, the individual CDR loops are clustered geometrically and the resulting cluster populations are plotted against each other to visualize and describe the simultaneous occurrence of distinct loop conformations (Fig. 2b). Figure 2b illustrates a matrix including all individual CDR loops and displays which loop conformations occur at the same time and therefore might be correlated. Strong correlations and multiple simultaneous occurring CDR loop conformations can be observed between the CDR-L1, CDR-L2, CDR-L3, CDR-H1, and CDR-H3 loops. A weaker correlation can be observed for the CDR-H2 loop. It only adopts two main conformations, which is limiting for the correlations with other CDR loops.

### Specific murine antigen-binding fragment binding to IL-18

**Fig. 3:**
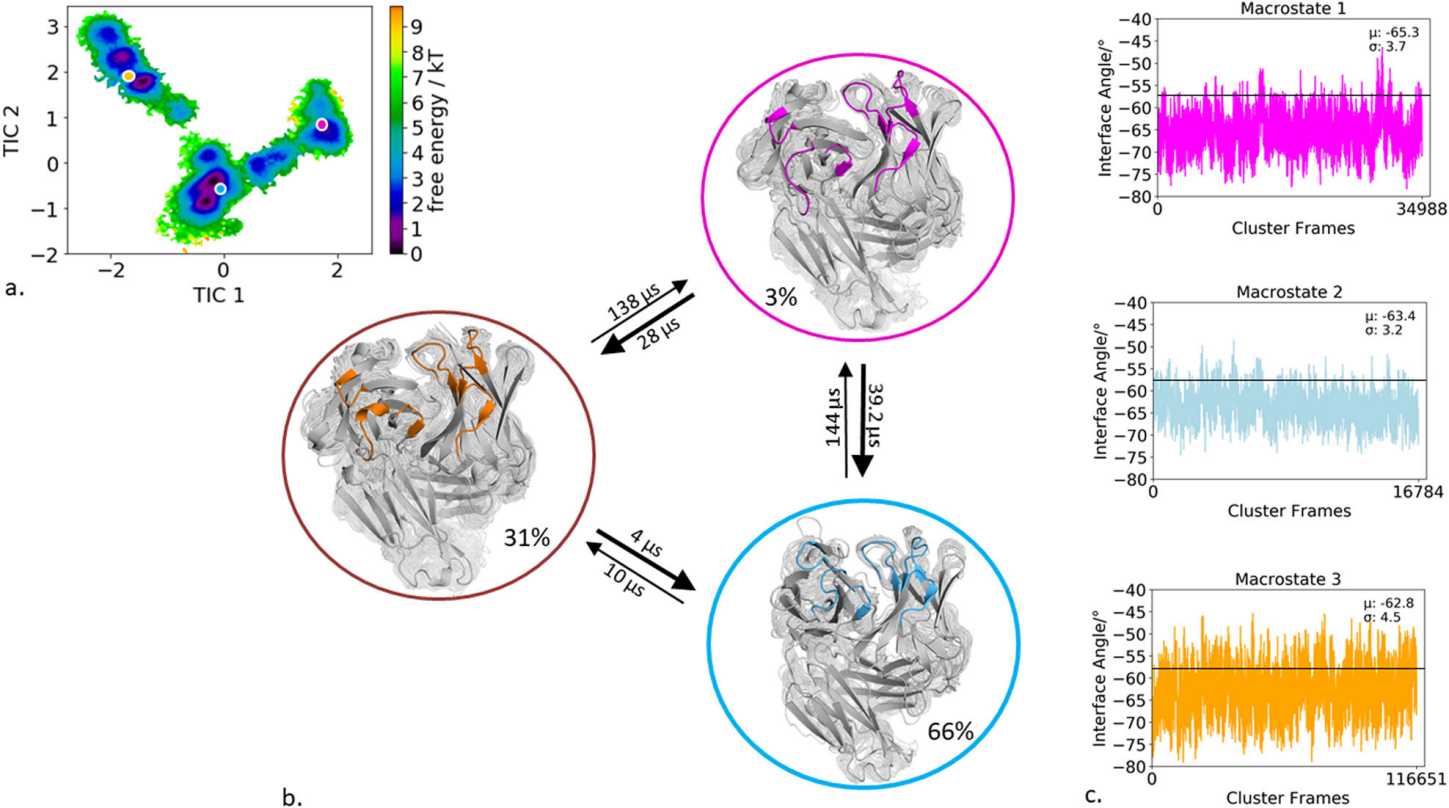
Paratope states of the specific IL-18 antibody including the results of the Markov-state model and shows the relative V_H_–V_L_ interface distributions for the respective macrostates. **a** Combined free energy landscape of all CDR loops with the respective three macrostate representatives. **b** Markov-state model, transition kinetics, and state probabilities of different paratope states for the specific IL-18 antibody. **c** Relative V_H_–V_L_ interface dynamics of the macrostate ensembles, color-coded according to the Markov-state model in (**b**). The horizontal line depicts the interface orientation of the crystal structure.

**Fig. 4:**
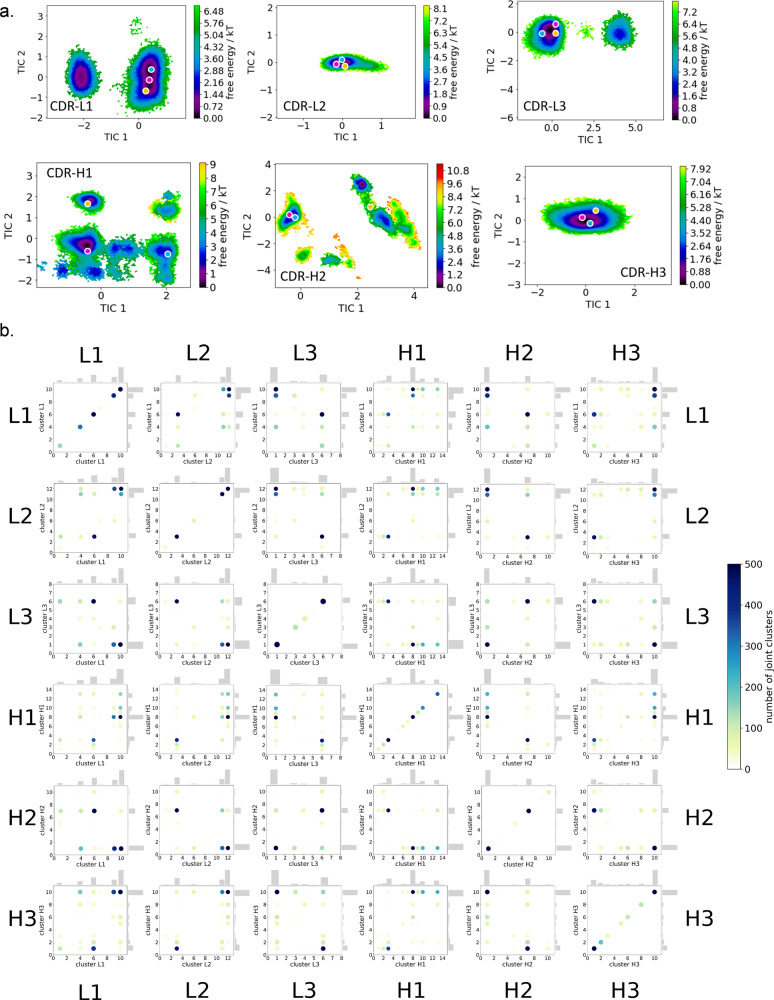
Free energy surfaces and loop correlations for all individual CDR loops, showing which loops contribute to conformational rearrangements in the different paratope states. **a** Free energy surfaces of all individual CDR loops of the IL-18 antibody including the projections of macrostates of Fig. 3b). **b** Visualization of simultaneous occurrence of CDR loop conformations for all CDR loops. We observe strong correlations between the different CDR loop conformations.

The second antibody studied, is the specific murine antigen-binding fragment (Fab) in complex with a the unique cytokine human IL-18, a proinflammatory cytokine that participates in the regulation of innate and acquired immunity (PDB accession code: 2VXT)^36^. Clustering of the bias-exchange simulation resulted in 174 cluster representatives, which were used as starting structures for each 100 ns of molecular dynamics simulations. Figure 3a illustrates the tICA free energy landscape of all CDR loops of the obtained 17.4 µs of trajectories. The Markov-state model in Fig. 3b reveals three macrostates, corresponding to three paratope states, including the state probabilities, transition timescales, and the respective macrostate ensembles. By using the macrostate representatives to analyze the influence of CDR loop states on the relative interdomain orientation we observe nearly no shifts in the distributions for the interface angle for different paratope states (Fig. 3c). Slightly bigger shifts upon conformational changes in the paratope can be observed for the elbow-angle. For the obtained three macrostates the elbow-angle distributions are not only shifted but reveal differences in distributions (Supplementary Fig. S1). To identify correlated loop movements, the free energy surfaces of the individual CDR loop states with the projected macrostate representatives of the overall paratope states are displayed in Fig. 4a.

Additionally, to the kinetic classification of the states, the individual CDR loops are clustered and the resulting cluster populations are plotted against each other to visualize and describe the simultaneous occurrence of distinct loop configurations (Fig. 4b). In this case, the CDR-H1 loop reveals strong correlated loop configurations with all other CDR loops.

### T-cell receptor-like Fab-Hyb3 in complex with the MAGE-A1-derived peptide

**Fig. 5:**
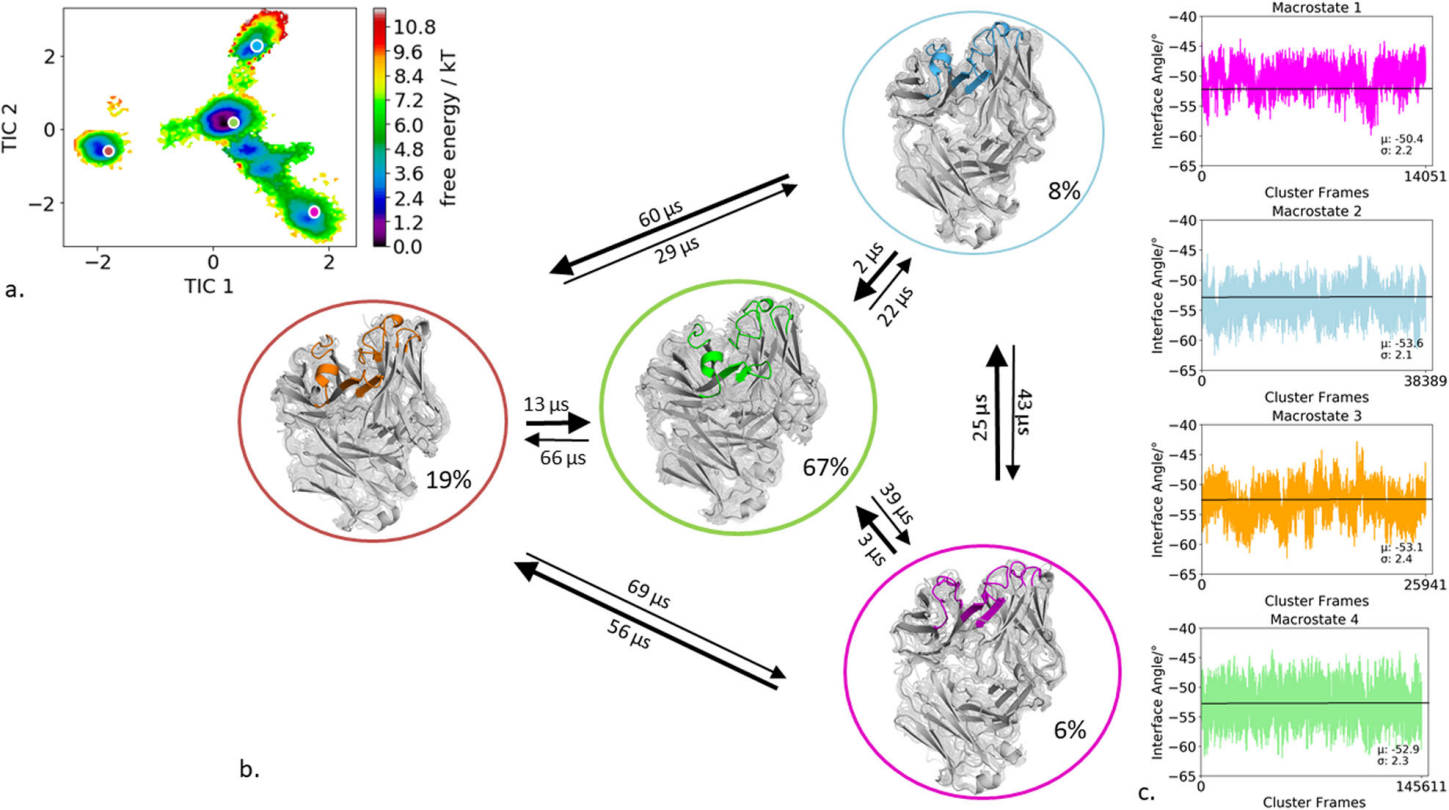
Paratope states of the matured Fab-Hyb3 including the results of the Markov-state model and shows the relative V_H_–V_L_ interface distributions for the respective macrostates. **a** Combined free energy landscape of all CDR loops with the respective four macrostate representatives. **b** Markov-state model, transition kinetics, and state probabilities of different paratope states for the matured Fab-Hyb3. **c** Relative V_H_–V_L_ interface dynamics of the macrostate ensembles, color-coded according to the Markov-state model in (**b**). The horizontal line depicts the interface orientation of the crystal structure.

**Fig. 6:**
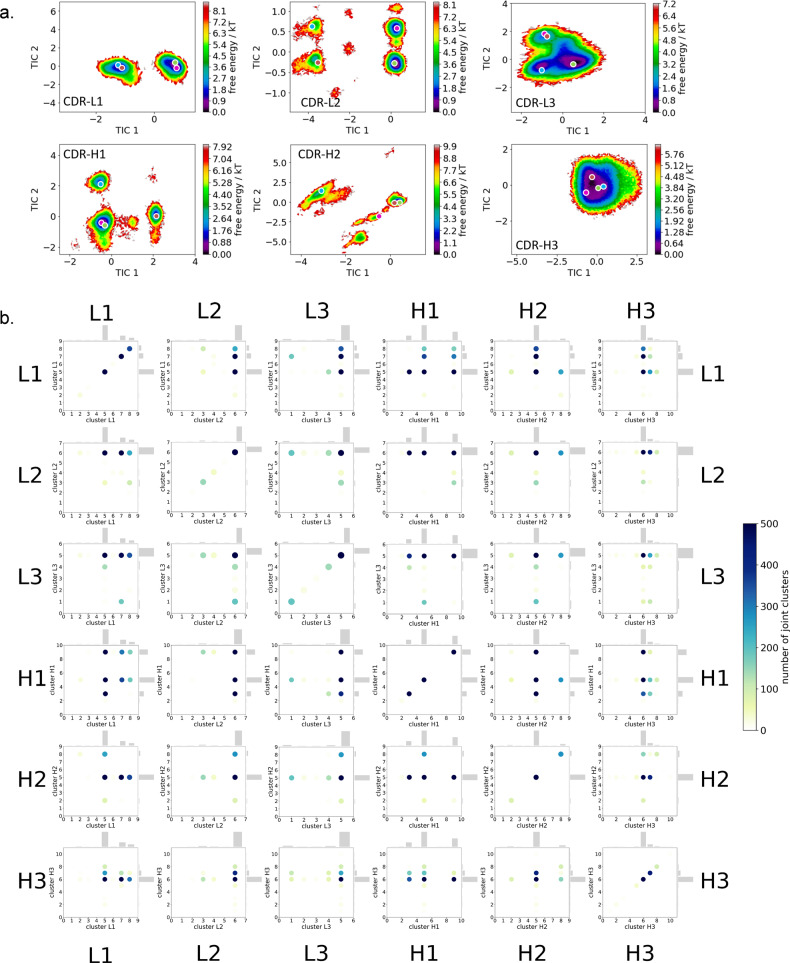
Free energy surfaces and loop correlations for all individual CDR loops, showing which loops contribute to conformational rearrangements in the different paratope states. **a** Free energy surfaces of all individual CDR loops of the Fab-Hyb3 including the projections of macrostates of Fig. 5b). **b** Visualization of simultaneous occurrence of CDR loop conformations for all CDR loops. We observe strong correlations between the different CDR loop conformations.

Another antibody fragment studied is the matured Fab-Hyb3 with T-cell receptor-like binding specificity, in complex with the melanoma antigen-encoding gene (MAGE)-A1-derived peptide (PDB accession code: 1W72)^37^. The free energy landscape and kinetic characterization of all CDR loops of the obtained 22.6 µs of trajectories are illustrated in Fig. 5a, b. The four macrostate ensembles were then used to investigate the role of the CDR loop binding interface on the relative V_H_ and V_L_ orientations. We observe small shifts in the V_H_ and V_L_ interdomain distributions upon conformational rearrangements in the paratope (Fig. 5c). In line with the small shift in the interface angle distributions also the elbow-angle distributions reveal a small shift upon conformational changes in the CDR loops (Supplementary Fig. S2). In line with the previous examples also in this case we observe strong correlations between the CDR loops and illustrate the simultaneous occurrence of specific CDR loop configurations in Fig. 6.

### Characterizing multispecificty of an SPE7 antibody

**Fig. 7:**
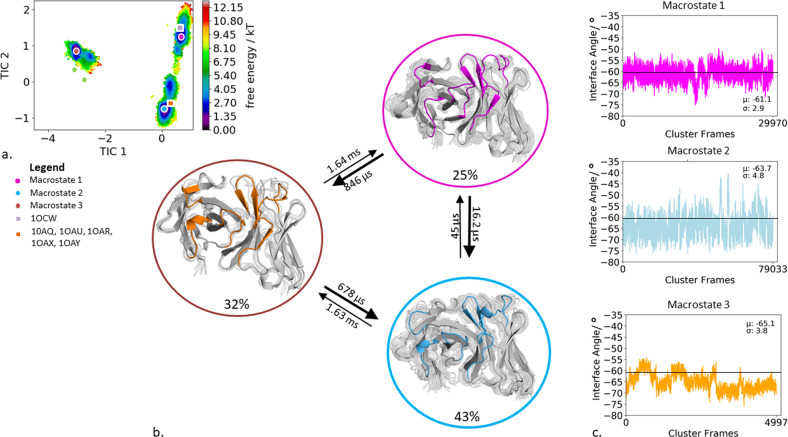
Paratope states of the SPE7 antibody including the results of the Markov-state model and shows the relative V_H_ − V_L_ interface distributions for the respective macrostates. **a** Combined free energy landscape of all CDR loops with the respective three macrostate representatives. **b** Markov-state model, transition kinetics, and state probabilities of different paratope states for the SPE7 antibody. **c** Relative V_H_–V_L_ interface dynamics of the macrostate ensembles, color-coded according to the Markov-state model in (**b**). The horizontal line depicts the interface orientation of the crystal structure.

The fourth antibody fragment investigated is part of a study characterizing multispecificity by conformational diversity, where the same SPE7 antibody can adopt various conformations and recognize different antigens (PDB accession code: 1OAQ)^38^. The free energy landscape of all CDR binding loops of 11.4 µs of trajectories reveals three accessible macrostates, in which all available crystal structures binding to different antigens are present (Fig. 7a). The available crystal structures reveal the greatest differences in the heavy chain, in particular the CDR-H3 loop (C*α*-RMSD of 5.38 Å between the 1OCW and 1OAX, C*α*-RMSD of 4.81 Å between the 1OCW and 1OAQ). The structure with the PDB accession code 1OCW, crystallized without antigen, shows the greatest structural difference, due to strong crystal packing effects in the CDR-H3, CDR-L3, and CDR-H1 loop^28^. While all other available structures lie in the same dominant minimum in solution, the 1OCW lies in a separate local side minimum. The three macrostate ensembles are again used to investigate if different CDR loop conformations reveal different relative V_H_ and V_L_ interdomain distributions (Fig. 7b). In line with the previous results, we observe shifted relative V_H_ and V_L_ interdomain distributions upon conformational changes in the binding interface. A substantial shift in the V_H_ and V_L_ interdomain distribution can be observed between the macrostates, in which the 1OCW and all the other available crystal structures are present (Fig. 7c). Also, for this antibody we observe a strong correlation and dependency of loop conformations for all CDR loops (Supplementary Fig. S5).

### Affinity maturation of an anti-lysozyme binding Fab

Another key aspect when characterizing different states of the complete binding interface is the affinity maturation process and its effect on the CDR loops. The binding site of polyreactive antibodies, which can bind with low affinity to various distinct antigens, has been shown to be substantially more flexible compared to matured antibodies^39,40^. To investigate the effect of affinity maturation on the accessible binding site states, the relative interdomain orientation and the elbow-angle, we chose the affinity maturation pair D44.1 (naïve) and the F10.6.6 (matured) anti-lysozyme binding Fab^41,42^. Both Fabs originate from the same germline rearrangement and are therefore related in sequence and structure. The D44.1 Fab differs from the matured F10.6.6 in twenty mutations, seven of them located in the CDR loops and four being directly in the V_H_–V_L_ interface. As starting structures for 200 ns bias-exchange simulations we used the Fabs with the PDB accession codes: 1MLC (naïve) and 1P2C (matured). By clustering the 200 ns bias-exchange simulations we already observe a substantial rigidification upon affinity maturation reflected in the number of resulting CDR binding site clusters. By using the same distance cut-off criterion of 1.2 Å as described in the “Methods” section the naïve antibody resulted in 301 cluster representatives while the matured antibody revealed only 141 clusters. The resulting 30.1 and 14.1 µs of trajectories were then used to construct a Markov-state model of the complete binding interface to test if the rigidification does not only occur in the CDR loops but also in the relative V_H_ and V_L_ interdomain distributions.

**Fig. 8:**
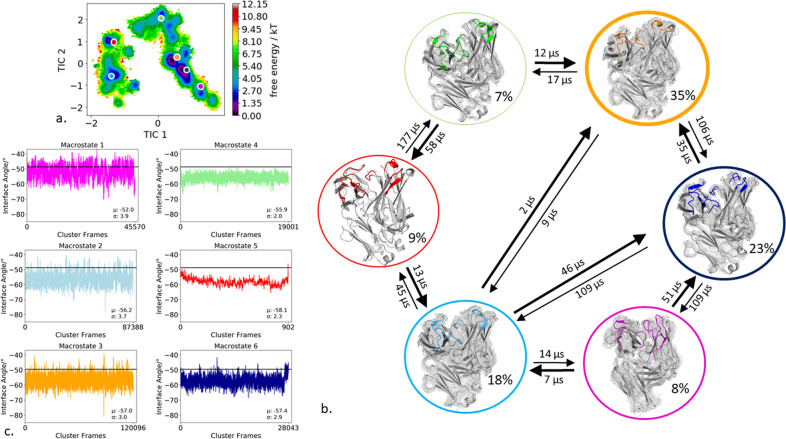
Paratope states of the naive D44.1 antibody including the results of the Markov-state model and shows the relative V_H_–V_L_ interface distributions for the respective macrostates. **a** Combined free energy landscape of all CDR loops with the respective six macrostate representatives. **b** Markov-state model, transition kinetics, and state probabilities of different paratope states for the naïve D44.1 antibody. **c** Relative V_H_–V_L_ interface dynamics of the macrostate ensembles, color-coded according to the Markov-state model in (**b**). The horizontal line depicts the interface orientation of the crystal structure.

Figure 8a shows the resulting free energy surface of the 30.1 µs of trajectories of the naïve antibody CDR binding interface. We obtained six macrostates with transition kinetics between the resulting six paratope states in the microsecond timescale (Fig. 8b). The V_H_ and V_L_ interdomain distributions of the six respective macrostate ensembles are shifted for the different CDR loop states, confirming a strong correlation of CDR loop rearrangements with the relative domain orientations (Fig. 8c). The relative interdomain orientations of the individual paratope macrostates for the D44.1 antibody fragment were computed by using the torsion-based interface angle definition and also by calculating the HL angle with ABangle (Supplementary Fig. 22). The relative interdomain distributions for the individual macrostates show the same trend and depict the same shifts upon conformational changes in the CDR loops independent of the applied method. The elbow-angle distributions for each macrostate ensemble are visualized in Supplementary Fig. S3 and reveal in this case an extremely low dependency on the distinct CDR loop states and the relative interdomain orientations. Very similar variations up to 55° for the elbow-angle distributions, as in the examples before, can be observed for the D44.1 antibody (Supplementary Fig. S3). The individual free energy surfaces of the CDR loops with the projected six macrostate representatives are shown in Supplementary Fig. S6a, while Supplementary Fig. S6b shows and visualizes the simultaneous occurrence of distinct loop configurations. Loop correlations can be observed between all CDR loops.

**Fig. 9:**
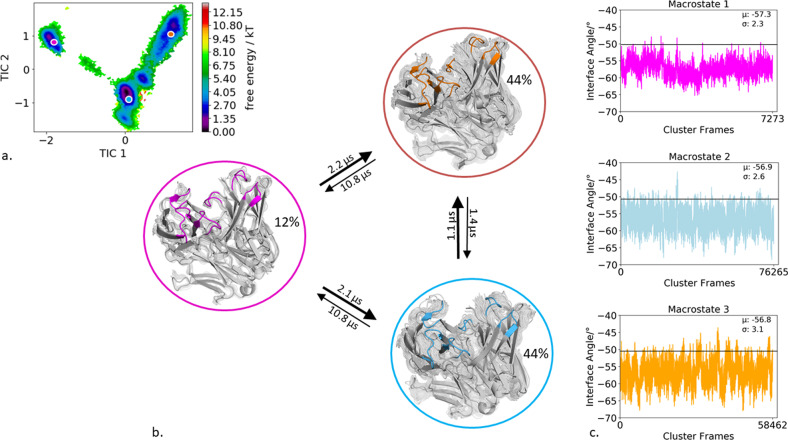
Paratope states of the matured F10.6.6 antibody including the results of the Markov-state model and shows the relative V_H_–V_L_ interface distributions for the respective macrostates. **a** Combined free energy landscape of all CDR loops with the respective three macrostate representatives. **b** Markov-state model, transition kinetics, and state probabilities of different paratope states for the matured F10.6.6 antibody. **c** Relative V_H_–V_L_ interface dynamics of the macrostate ensembles, color-coded according to the Markov-state model in (**b**). The horizontal line depicts the interface orientation of the crystal structure.

The free energy surface of CDR binding loops of the matured F10.6.6 antibody (14.1 µs of trajectories) is illustrated in Fig. 9a. The Markov-state model in Fig. 9b reveals three macrostates with conformational transitions between different CDR binding site states in the micro-to millisecond timescales. Again, by analyzing the respective ensemble of each macrostate to identify the role of conformational changes in the paratope on the interface angle, we observe nearly no difference in the relative interdomain orientations between the different paratope states (Fig. 9c). Also, in the elbow-angle distributions nearly no shifts can be observed for different CDR loop states (Supplementary Fig. S4). However, upon affinity maturation the elbow angle substantially rigidifies compared to the naïve antibody D44.1 antibody and only reveals variations up to 40°. Supplementary Fig. S7a, S7b shows again the free energy landscapes of the individual CDR loops and visualize the simultaneous occurrence of loop conformations. We observe a strong correlation and dependency of loop conformations for the frames (in particular for macrostate 2) in Supplementary Fig. S7a for the CDR-L1, CDR-L2, CDR-H1, CDR-H2, and CDR-H3 loop conformations.

To visualize the conformational diversity of each CDR loop individually we included 2D-rmsd plots, residual b-factors and ensemble pictures for each macrostate in the supporting information (Supplementary Figs. S10–S20).

## Discussion

Antibody CDR loops are flexible and can adopt various distinct conformations^31–33,38^. Studies revealed that the high conformational diversity of the CDR loops can be captured by kinetically characterizing the loops as ensembles in solution. For all CDR loops conformational transitions between canonical clusters and additional dominant solution structures have been observed^18,27,28^. Additionally, these conformational transitions between different CDR loop states occur in the micro-to-millisecond timescale^18,27,28,43^, while the relative V_H_ and V_L_ interdomain dynamics can be captured in the 0.1–10 ns timescale^44,45^.

Our results are in line with previous studies; however, we observe that the slow components of these relative V_H_ and V_L_ interdomain dynamics, slower than 10 ns, might be strongly correlated with the CDR loop rearrangements occurring in the micro-to millisecond timescale. Our results reveal that certain CDR loop conformations favor specific interface angle distributions, fluctuating in the 0.1–10 ns timescale. Similar fast dynamics as for the relative V_H_ and V_L_ interdomain dynamics can be seen for the elbow angle, which exhibits the majority of movements in the low nanosecond timescale. It has already been shown that the elbow-angle has high flexibility and that antigen-binding or even single point-mutations can strongly influence the elbow-angle^35,36,46^. In all examples, we observe broader variations in the elbow-angle compared to the relative V_H_ and V_L_ interdomain dynamics. This is line with the idea that conformational CDR loop rearrangements, which are involved in the antigen-binding process, induce shifts or changes in the elbow-angle distributions. Thus, also in this case the transitions between different CDR loop binding interface states, which strongly influence the relative V_H_ and V_L_ interdomain dynamics, might also correlate with the slower dynamics of the elbow-angle movements. Our results clearly show that conformational rearrangements in the CDR loop backbone occur in the micro-to-millisecond timescale, while the interdomain and elbow-angle dynamics can be captured in the low nanosecond timescale. Side-chain flexibility (vibrations and side-chain rotation) occurs on a substantially faster timescale (in the low nanosecond timescale), compared to conformational changes in the backbone (in the microsecond timescale)^47–50^. Thus, for all our macrostates possible side-chain conformations and their dynamics should be represented comprehensively in our simulations.

Especially interesting is the effect of affinity maturation on the resulting CDR binding loop states and the resulting changes in the distributions of the relative V_H_ and V_L_ interdomain dynamics and the elbow angle. The results of the naïve and matured antibody in Figs. 8b and 9b, respectively, indicate that not only the paratope rigidifies upon affinity maturation, but also the relative V_H_ and V_L_ interdomain dynamics and the elbow-angle exhibit less flexibility (Figs. 8c, 9c, Supplementary Figs. S3, S4). The direct comparison of the conformational spaces between the naïve and the matured Fab in the same coordinate system is shown in Supplementary Fig. S8 and a substantial decrease in conformational space is evident. The D44.1 antibody fragment was crystallized with and without antigen (PDB accession codes: 1MLC, 1MLB, respectively). We observe that the X-ray structure of the D44.1 antibody crystallized without antigen lies in a local side minimum due to crystal packing effects, while the bound structure belongs to the dominant minimum in solution^28^. Also the relative interdomain orientation V_H_–V_L_ differs 2° between the two available X-ray structures and both interface orientations are present within the obtained ensemble in solution. For the naïve D44.1 antibody, conformational rearrangements of the paratope result in substantial shifts of the relative interface orientation. The sampled canonical clusters for the individual CDR loops, for both the naïve and the matured antibody fragment, are depicted in Supplementary Tables S4 and S5. The results clearly reveal that various canonical cluster representatives lie within the same minimum in solution and that we clearly see transitions between different canonical structures. A substantial rigidification upon maturation of the binding interface loops can be observed, which is in line with the smaller variability of the V_H_ and V_L_ interdomain dynamics and the reduced elbow-angle flexibility. Smaller conformational changes in binding site loops also induce less effect on the interface angle and the elbow-angle distributions. In line with these observations, also the matured Fab-Hyb3 exhibits only small shifts in the relative V_H_ and V_L_ interdomain angles and elbow-angle distributions for the different paratope macrostates in solution. Supplementary Table S3 illustrates the sampled canonical clusters and to shows which macrostate in solution they belong to. Again, various canonical clusters belong to the same kinetic minimum in solution. Especially interesting is that the Fab-Hyb3 consists of a lambda light chain and is the representative canonical cluster median of the L1-11-3 cluster, containing only lambda chain structures. The other CDR-L1 loop canonical clusters are not present within the obtained ensemble in solution, indicating that the kappa CDR-L1 loop conformations might not be energetically favorable when starting from a lambda light chain CDR-L1 conformation. Besides sampling various canonical clusters for all CDR loops, we also observe a strong correlation between different CDR loop conformational rearrangements (Fig. 6).

In agreement with previous findings for highly specific antibody fragments, the IL-18 specific Fab shows only small shifts in the relative interdomain angle distributions upon conformational changes in the CDR loop states between the three different macrostates (Fig. 3, Fig. 4). Additionally, Fig. 4 displays the correlation between different CDR loops, in particular, the CDR-H1 loop appears to strongly influence the CDR-H3, CDR-H2, CDR-L3, and CDR-L2 loop and thereby consequently reduces the combinatorics of possible binding site conformations. Furthermore, we identified for each individual loop that various canonical clusters are sampled within our CDR loop ensemble in solution and the assignments to the respective macrostates are shown in Supplementary Table S2. The substantial shifts in the elbow-angle distributions can be interpreted as allosteric effect induced by conformational changes in the CDR loops, which is in line with previously shown results, discussing substantial changes in the elbow-angle upon antigen binding^34,35^. Another example, where the different CDR loop conformational states strongly influence the resulting relative V_H_ and V_L_ interdomain dynamics is the human anti-polyhydroxybutyrate antibody variable fragment. The obtained four paratope states show substantial differences in the interface angle distributions upon differences in the CDR loop conformations, following the idea of certain CDR loop states favoring certain V_H_ and V_L_ interdomain orientations (Fig. 1). Furthermore, the free energy landscape in Fig. 1a shows a strong state separation in the first tIC component, dominated by correlated structural rearrangements in the CDR-H3, CDR-H1, CDR-L1, and CDR-L2 loops. Supplementary Table S1 summarizes the individual CDR loops which canonical clusters are sampled and even belong to the same macrostate in solution. Within macrostate 4, which is the highest populated paratope state, a high number of the available canonical cluster representatives for all CDR loops are present, this especially is true for the CDR-L3 loop. Various canonical clusters are present within the obtained ensembles in solution and again belong to the same kinetic minimum and thus should be combined.

Multispecificity of antibodies has been discussed to be mediated by conformational diversity. Conformational diversity follows the concept that one single sequence can adopt multiple structures and functions. Thus the functional diversity of the limited repertoire of antibody sequences is increased and the evolution of new proteins and functions is facilitated^31–33^.

The variable fragment of SPE7 was crystallized with four different antigens and without antigens. In the absence of the antigen, substantial conformational rearrangements due to crystal packing effects, especially in the CDR-H3, CDR-H1, and CDR-L3 loops can be observed. While five of the six structures belong to the same kinetic dominant minimum in solution, the 1OCW, crystallized without antigen, differing substantially from all other structures, lies in a side minimum in solution. We obtained three macrostates and again observe shifts in the interface angle distributions upon changes in the binding site CDR loop conformations. Furthermore, Supplementary Table S6 summarizes the canonical cluster representatives present within the obtained macrostate ensembles in solution and again for certain CDR loops various canonical cluster representatives belong to the same kinetic minimum and thus should be combined. For the highest populated canonical cluster of the CDR-L3 loop with a loop length of nine, five of the six available canonical clusters belong to the same kinetic minimum. Our results are in strong agreement with previous studies, showing that the CDR-L3 loop can adopt various conformations in solution, in which the majority of canonical clusters are present and even belong to the same kinetic minimum^27^. Recent studies discussed the hidden role of the DE loops in antibodies, which are usually considered as framework region. This fourth solvent-exposed loop, the so-called DE loop, adjacent to CDR1 and CDR2 loops, also shows, similar as the CDR loops, a high variability in sequence and structure and is involved in some antibody-antigen interactions; however, even if no direct contact with the antigen is present, it can modulate the behavior of the CDR loops^51^. The DE loop located on the heavy chain is called H4 loop, while the DE loop of the light chain is known as L4 loop. The crucial role of the H4 binding site loop for molecular recognition and in the antigen-binding process has recently been discussed for T-cell receptors^52^. The free energy surfaces and conformational diversity of both the L4 and the H4 loops are illustrated in Supplementary Fig. S9. We clearly see that both the L4 and H4 loops are correlated with the CDR loops, indicating their prominent role in antigen-binding and their influence on the binding site loop conformations.

**Fig. 10:**
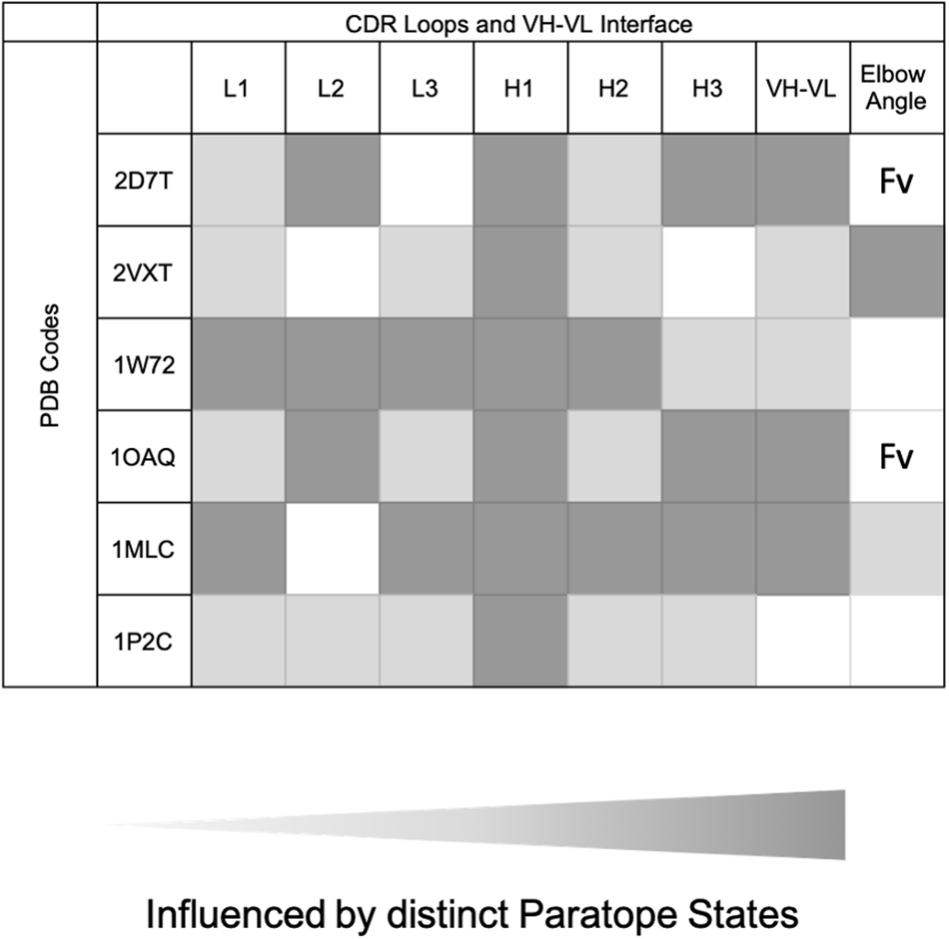
Overview and summary of all investigated antibody fragments with their respective PDB codes, visualizing which of the CDR loops is influenced by different paratope states. As we observe a strong dependency of the CDR loop conformations on the relative V_H_–V_L_ domain orientation we also included the influence of different paratope states on the relative interface distributions. The strength of the influence of different paratope states on the individual CDR loops, the V_H_–V_L_ domain orientation and the elbow-angle correlates with the intensity of the different shades of gray.

For antibody structure design our results imply, that all CDR loops can adopt different conformations in solution, which are not only strongly correlated with each other, but can also shift the relative V_H_–V_L_ interdomain distributions. Conformational changes in the paratope are mainly dominated by CDR-L2, CDR-H1, and CDR-H3 loop rearrangements, which directly influence the relative interdomain orientations and in some cases even the elbow-angle (Fig. 10). Conformational changes in the antibody binding site can induce changes in the elbow-angle, indicating an allosteric effect. The CDR-H2 loop conformation has been discussed to be determined by the heavy chain framework residue 71^53^. In two studied antibody fragments residue 71 is an arginine resulting in a stabilization of the CDR-H2 and CDR-H1 loop, as salt bridges and hydrogen bond interactions are formed. While the salt bridges and hydrogen bond interactions with the CDR-H2 loop occur in up to 90% of the frames, interactions with the CDR-H1 loop occur only in certain macrostates, indicating that residue H71 plays an important role in stabilizing and favoring specific CDR-H1 loop conformational states in solution. Besides interactions with residue H71, both the CDR-H1 and CDR-H2 loops are strongly influenced by rearrangements of the paratope. The CDR-H3 loop is not influenced by different paratope states in the example of the specific IL-18 binding antibody. Apart from the high specificity of this antibody, the CDR-H3 loop is rather short, containing only four residues. Both aspects together might explain this low flexibility and the small contributions to conformational rearrangements of the paratope. However, it has already been shown, that a less specific or naïve antibody of the same short CDR-H3 loop length, can reveal a high flexibility, possibly influencing the other CDR loop conformations^18^. In line with these observations, the multispecific SPE7 antibody contains a nine residue long CDR-H3 loop, which substantially contributes together with the CDR-H1 and CDR-L2 loops to rearrangements of the CDR loop binding interface. The CDR loop length might influence the resulting flexibility, but as can be seen in the affinity maturation pair, the CDR loops of the same length rigidify substantially upon affinity maturation, indicating that the conformational diversity of a loop and the influence on the other binding CDR loops is governed by their sequence composition and not necessarily by their length. Upon affinity maturation and for highly specific antibodies we observe smaller conformational changes in the CDR loops and consequently also smaller shifts in the relative V_H_–V_L_ orientation, compared to naïve or multispecific antibodies. Additionally, we sample conformational transitions between different canonical clusters for all CDR loops and identify additional dominant solution structures, which are not apparent from X-ray structures most likely due to crystal packing effects. We also observe that various canonical conformations belong to the same kinetic minimum in solution and thus might be combined.

In conclusion, antibody paratopes are flexible and exist in several different conformations. We characterize kinetically accessible paratope states and observe correlated binding interface loop rearrangements for several antibody fragments. Besides kinetic characterization of the paratope into different correlated CDR loop states, we also present a strong dependence of the CDR loop conformations on the relative interdomain orientation. In the studied Fab cases we also see an influence of the different paratope states, including CDR loop movements and changes in the relative interdomain orientation, on the obtained elbow-angle distributions. We show that antibody CDR loop conformations are strongly correlated, reducing the combinatorics of possible states of the complete binding interface and shifting the relative V_H_ and V_L_ orientation and the elbow-angle distributions. These findings have broad implications in the field of antibody design and in the development of biotherapeutics as they provide a new paradigm in the understanding of CDR binding loop states, antibody-antigen recognition, relative V_H_ and V_L_ interface angles and elbow-angle distributions and their respective dynamics.

## Methods

### Structure preparation

Experimental structure information was available for all considered antibody fragments. We deleted the co-crystallized antigen in all complex crystal structures. The starting structures for simulations were prepared in MOE (Molecular Operating Environment, Chemical Computing Group, version 2018.01) using the Protonate3D tool^54,55^. To neutralize the charges we used the uniform background charge^56–58^. Using the tleap tool of the AmberTools18^56,57^ package, the crystal structures were soaked with cubic water boxes of TIP3P water molecules with a minimum wall distance of 10 Å to the protein^59^. The box size in MD simulations can influence the resulting dynamics if sampling is insufficient^60,61^. For all crystal structures parameters of the AMBER force field 14SB were used^62^. The antibody fragments were carefully equilibrated using a multistep equilibration protocol^63^.

### Metadynamics simulations

To enhance the sampling of the conformational space well-tempered bias-exchange metadynamics^64–66^ simulations were performed in GROMACS^67,68^ with the PLUMED 2 implementation^69^. As enhanced sampling technique, we chose metadynamics as it allows to focus the enhanced sampling on predefined collective variables (CV). The sampling is accelerated by a history-dependent bias potential, which is constructed in the space of the CVs^64,66,70^. As collective variables, we used a well-established protocol, boosting a linear combination of sine and cosine of the ψ torsion angles of all CDR loops calculated with functions MATHEVAL and COMBINE implemented in PLUMED 2^18,27,43,52,69,71^. As discussed previously the ψ torsion angle captures conformational transitions comprehensively^72^. The underlying method presented in this paper has been validated in various studies against a large number of experimental results. The simulations were performed at 300 K in an NpT ensemble using the GPU implementation of the pmemd module^47^ to be as close to the experimental conditions as possible and to obtain the correct density distributions of both protein and water. We used a Gaussian height of 10.0 kcal/mol. Gaussian deposition occurred every 1000 steps and a bias factor of 10 was used. 200 ns of bias-exchange metadynamics simulations were performed for each available antibody fragment crystal structure. The resulting trajectories were clustered in cpptraj^57,73^ by using the average linkage hierarchical clustering algorithm with a distance cut-off criterion of 1.2 Å resulting in a large number of clusters. The cluster representatives for the antibody fragments were equilibrated and simulated for 100 ns using the AMBER18^74^ simulation package.

### Molecular dynamics simulations

Molecular dynamics simulations were performed in an NpT ensemble using pmemd.cuda^47^. Bonds involving hydrogen atoms were restrained by applying the SHAKE algorithm^75^, allowing a time step of 2.0 fs. Atmospheric pressure (1 bar) of the system was set by weak coupling to an external bath using the Berendsen algorithm^76^. The Langevin thermostat^77^ was used to maintain the temperature during simulations at 300 K.

With the obtained trajectories we performed a time-lagged independent component analysis (tICA) using the python library PyEMMA 2 employing a lag time of 10 ns^78^. Thermodynamics and kinetics were calculated with a Markov-state model^79^ by using PyEMMA 2, which uses the k-means clustering algorithm^80^ to define microstates and the PCCA + clustering algorithm^81^ to coarse grain the microstates to macrostates. Markov-state models are network models which provide valuable insights for conformational states and transition probabilities between them, as it is possible to accurately identify the boundaries between two states^79^. The states are defined based on kinetic criteria, which allow us to identify the boundaries between free energy wells. Basically, MSMs coarse-grain the system’s dynamics, which reflect the free energy surface and ultimately determine the system’s structure and dynamics. Thus, MSMs provide important insights and enhance the understanding of states and transition probabilities, which can make a quantitative connection with experiment^79,82^.

The sampling efficiency and the reliability of the Markov-state model (e.g., defining optimal feature mappings) can be evaluated with the Chapman–Kolmogorov test^46,83^, by using the variational approach for Markov processes^84^ and by taking into account the fraction of states used, as the network states must be fully connected to calculate probabilities of transitions and the relative equilibrium probabilities. To build the Markov-state model we used the backbone torsions of the respective CDR loop, defined 150 microstates using the k-means clustering algorithm and applied a lag time of 10 ns.

The median canonical structure information for each CDR loop, provided in the tables in the supplementary material, was extracted from the PyIgClassify database^22^ and compared to the obtained CDR loop ensembles in solution. We then used the respective macrostate ensembles to investigate correlations between the different paratope states and the relative V_H_ and V_L_ orientations and the elbow angles.

### Relative V_H_ and V_L_ orientations and elbow-angle calculations

For the relative V_H_ and V_L_ orientations, described in this study, we defined a torsion angle between the center of mass (COM) of the CDR loops of the light chain, the COM of the V_L_ domain, the COM of the V_H_ domain and the COM of the CDR loops of the heavy chain. Additionally, we also performed calculations using the well-established program ABangle^85,86^ to calculate the dependence of the relative V_H_–V_L_ orientations on the CDR loop conformations. The results of the ABangle are shown in Supplementary Table S8. A comparison of the ABangle results with our defined torsion angle to characterize the antibody interface is illustrated in Supplementary Fig. 22.

As measure for the elbow-angle we calculated a torsion angle between the COM of variable domain, a defined vector between the COMs of the switch regions and the COM of the constant region. We also included the results (Supplementary Table S7) for the elbow-angle calculations by using the measure presented by Stanfield et al. in the supporting information^87^.

### Reporting summary

Further information on research design is available in the Nature Research Reporting Summary linked to this article.

## Supplementary information

Supplementary Information

Reporting Summary

## Data Availability

All data generated or analyzed during this study are included in this published article (and its supplementary information files).

## Code availability

The code generated used in the current study is available from the corresponding author on reasonable request.

## References

[1] Kaplon H, Reichert JM (2019). Antibodies to watch in 2019. mAbs.

[2] Reichert JM (2016). Antibodies to watch in 2017. mAbs.

[3] Chames P, Van Regenmortel M, Weiss E, Baty D (2009). Therapeutic antibodies: successes, limitations and hopes for the future. Br. J. Pharm..

[4] Kaplon H, Muralidharan M, Schneider Z, Reichert JM (2020). Antibodies to watch in 2020. mAbs.

[5] Nguyen MN, Pradhan MR, Verma C, Zhong P (2017). The interfacial character of antibody paratopes: analysis of antibody–antigen structures. Bioinformatics.

[6] Davies DR, Chacko S (1993). Antibody structure. Acc. Chem. Res..

[7] Wong, W. K., Leem, J., Deane, C. M. Comparative analysis of the CDR loops of antigen receptors. *bioRxiv*10.1101/709840 (2019). 10.3389/fimmu.2019.02454 PMC6803477 31681328

[8] Al-Lazikani B, Lesk AM, Chothia C (1997). Standard conformations for the canonical structures of immunoglobulins11Edited by I. A. Wilson. J. Mol. Biol..

[9] Chothia C (1989). Conformations of immunoglobulin hypervariable regions. Nature.

[10] North B, Lehmann A, Dunbrack RL, New A (2011). Clustering of antibody CDR loop conformations. J. Mol. Biol..

[11] Martin ACR, Thornton JM (1996). Structural families in loops of homologous proteins: automatic classification, modelling and application to antibodies. J. Mol. Biol..

[12] Regep C, Georges G, Shi J, Popovic B, Deane CM (2017). The H3 loop of antibodies shows unique structural characteristics. Proteins.

[13] Burkovitz A, Sela-Culang I, Ofran Y (2014). Large-scale analysis of somatic hypermutations in antibodies reveals which structural regions, positions and amino acids are modified to improve affinity. FEBS J..

[14] Davenport TM (2016). Somatic hypermutation-induced changes in the structure and dynamics of HIV-1 broadly neutralizing antibodies. Structure.

[15] Clark LA, Ganesan S, Papp S, van Vlijmen HWT (2006). Trends in antibody sequence changes during the somatic hypermutation process. J. Immunol..

[16] Bassing CH, Swat W, Alt FW (2002). The mechanism and regulation of chromosomal V(D)J recombination. Cell.

[17] French D, Laskov R, Scharff M (1989). The role of somatic hypermutation in the generation of antibody diversity. Science.

[18] Fernández-Quintero ML (2019). Characterizing the diversity of the CDR-H3 loop conformational ensembles in relationship to antibody binding properties. Front. Immunol..

[19] Marks C (2017). Sphinx: merging knowledge-based and ab initio approaches to improve protein loop prediction. Bioinformatics.

[20] Kuroda D, Shirai H, Kobori M, Nakamura H (2009). Systematic classification of CDR-L3 in antibodies: Implications of the light chain subtypes and the VL–VH interface. Proteins: Struct., Funct., Bioinform..

[21] Townsend CL (2016). Significant differences in physicochemical properties of human immunoglobulin kappa and lambda CDR3 Regions. Front Immunol..

[22] Adolf-Bryfogle J, Xu Q, North B, Lehmann A, Dunbrack RL (2015). PyIgClassify: a database of antibody CDR structural classifications. Nucleic Acids Res..

[23] Marcatili P, Rosi A, Tramontano A (2008). PIGS: automatic prediction of antibody structures. Bioinformatics.

[24] Weitzner BD (2017). Modeling and docking of antibody structures with Rosetta. Nat. Protoc..

[25] Kuroda D, Shirai H, Jacobson MP, Nakamura H (2012). Computer-aided antibody design. Protein Eng., Des. Selection.

[26] Nowak J (2016). Length-independent structural similarities enrich the antibody CDR canonical class model. mAbs.

[27] Fernández-Quintero ML, Math BF, Loeffler JR, Liedl KR (2019). Transitions of CDR-L3 loop canonical cluster conformations on the micro-to-millisecond timescale. Front. Immunol..

[28] Fernández-Quintero ML, Kraml J, Georges G, Liedl KR (2019). CDR-H3 loop ensemble in solution – conformational selection upon antibody binding. mAbs.

[29] Abhinandan KR, Martin ACR (2010). Analysis and prediction of VH/VL packing in antibodies. Protein Eng. Des. Selection.

[30] Banfield MJ, King DJ, Mountain A, Brady RL (1997). VL:VH domain rotations in engineered antibodies: crystal structures of the Fab fragments from two murine antitumor antibodies and their engineered human constructs. Proteins: Struct. Funct., Bioinforma..

[31] James LC, Tawfik DS (2003). Conformational diversity and protein evolution—a 60-year-old hypothesis revisited. Trends Biochem. Sci..

[32] Pauling L (1940). A theory of the structure and process of formation of antibodies*. J. Am. Chem. Soc..

[33] Foote J, Milstein C (1994). Conformational isomerism and the diversity of antibodies. Proc. Natl Acad. Sci. USA.

[34] Sotriffer CA, Rode BM, Varga JM, Liedl KR (2000). Elbow flexibility and ligand-induced domain rearrangements in antibody Fab NC6.8: large effects of a small hapten. Biophys. J..

[35] Sotriffer CA, Liedl KR, Linthicum DS, Rode BM, Varga JM (1998). Ligand-induced domain movement in an antibody fab: molecular dynamics studies confirm the unique domain movement observed experimentally for fab NC6.8 upon complexation and reveal its segmental flexibility11Edited by I. Wilson. J. Mol. Biol..

[36] Argiriadi MA, Xiang T, Wu C, Ghayur T, Borhani DW (2009). Unusual water-mediated antigenic recognition of the proinflammatory cytokine interleukin-18. J. Biol. Chem..

[37] Hülsmeyer M (2005). A major histocompatibility complex·peptide-restricted antibody and T cell receptor molecules recognize their target by distinct binding modes: crystal structure of human leukocyte antigen (HLA)-A1·MAGE-A1 in complex with FAB-HYB3. J. Biol. Chem..

[38] James LC, Roversi P, Tawfik DS (2003). Antibody multispecificity mediated by conformational diversity. Science.

[39] Zhou Z-H, Tzioufas AG, Notkins AL (2007). Properties and function of polyreactive antibodies and polyreactive antigen-binding B cells. J. Autoimmun..

[40] Gunti S, Notkins AL (2015). Polyreactive antibodies: function and quantification. J. Infect. Dis..

[41] Braden BC (1994). Three-dimensional structures of the free and the antigen-complexed Fab from monoclonal anti-lysozyme antibody D44.1. J. Mol. Biol..

[42] Cauerhff A, Goldbaum FA, Braden BC (2004). Structural mechanism for affinity maturation of an anti-lysozyme antibody. Proc. Natl Acad. Sci. USA.

[43] Fernández-Quintero, M. L., Heiss, M. C., Liedl, K. R. Antibody humanization—the Influence of the antibody framework on the CDR-H3 loop ensemble in solution. *Protein Engineering, Design and Selection*10.1093/protein/gzaa004 (2020). 10.1093/protein/gzaa004 PMC7098879 32129452

[44] Fernández-Quintero, M. L. et al. V_H_-V_L_ interdomain dynamics observed by computer simulations and NMR. *Proteins: Struct. Funct. Bioinform.*10.1002/prot.25872 (2020). 10.1002/prot.25872 PMC7317758 31904133

[45] Niederfellner G (2011). Epitope characterization and crystal structure of GA101 provide insights into the molecular basis for type I/II distinction of CD20 antibodies. Blood.

[46] Karush J (1961). On the Chapman–Kolmogorov Equation. Ann. Math. Stat..

[47] Salomon-Ferrer R, Götz AW, Poole D, Le Grand S, Walker RC (2013). Routine microsecond molecular dynamics simulations with AMBER on GPUs. 2. Explicit solvent particle mesh Ewald. J. Chem. Theory Comput..

[48] Fuchs JE (2015). Independent metrics for protein backbone and side-chain flexibility: time scales and effects of ligand binding. J. Chem. Theory Comput..

[49] Boehr DD, Dyson HJ, Wright PE (2006). An NMR perspective on enzyme dynamics. Chem. Rev..

[50] Henzler-Wildman K, Kern D (2007). Dynamic personalities of proteins. Nature.

[51] Kelow, S. P., Adolf-Bryfogle, J., Dunbrack, R. L. Hiding in plain sight: structure and sequence analysis reveals the importance of the antibody DE loop for antibody-antigen binding. *bioRxiv*10.1101/2020.02.12.946350 (2020). 10.1080/19420862.2020.1840005 PMC7671036 33180672

[52] Fernández-Quintero ML, Seidler CA, Liedl KR (2020). T-Cell receptor variable β domains rigidify during affinity maturation. Sci. Rep..

[53] Tramontano A, Chothia C, Lesk AM (1990). Framework residue 71 is a major determinant of the position and conformation of the second hypervariable region in the VH domains of immunoglobulins. J. Mol. Biol..

[54] Labute P (2009). Protonate3D: assignment of ionization states and hydrogen coordinates to macromolecular structures. Proteins.

[55] Chemical Computing Group (CCG), 1010 Sherbrooke St. West, Suite #910, Montreal, QC, Canada, H3A 2R7 (2020).

[56] Case DA (2016). AMBER 2016.

[57] Roe DR, Cheatham TE (2013). PTRAJ and CPPTRAJ: software for processing and analysis of molecular dynamics trajectory data. J. Chem. Theory Comput..

[58] Hub JS, de Groot BL, Grubmüller H, Groenhof G (2014). Quantifying artifacts in ewald simulations of inhomogeneous systems with a net charge. J. Chem. Theory Comput..

[59] Jorgensen WL, Chandrasekhar J, Madura JD, Impey RW, Klein ML (1983). Comparison of simple potential functions for simulating liquid water. J. Chem. Phys..

[60] Gapsys, V., de Groot, B. L. Comment on “Valid molecular dynamics simulations of human hemoglobin require a surprisingly large box size.” *bioRxiv*10.1101/563064 (2019). 10.7554/eLife.44718 PMC6586461 31219782

[61] El Hage K, Hédin F, Gupta PK, Meuwly M, Karplus M (2018). Valid molecular dynamics simulations of human hemoglobin require a surprisingly large box size. eLife.

[62] Maier JA (2015). ff14SB: Improving the accuracy of protein side chain and backbone parameters from ff99SB. J. Chem. Theory Comput..

[63] Wallnoefer HG, Liedl KR, Fox T (2011). A challenging system: free energy prediction for factor Xa. J. Comput. Chem..

[64] Barducci A, Bussi G, Parrinello M (2008). Well-tempered metadynamics: a smoothly converging and tunable free-energy method. Phys. Rev. Lett..

[65] Biswas, M., Lickert, B. Stock, G. *Metadynamics Enhanced Markov Modeling of Protein Dynamics*. 10.1021/acs.jpcb.7b11800 (2018). 10.1021/acs.jpcb.7b11800 29338243

[66] Barducci A, Bonomi M, Parrinello M (2011). Metadynamics. WIREs Comput Mol. Sci..

[67] Abraham MJ (2015). GROMACS: High performance molecular simulations through multi-level parallelism from laptops to supercomputers. SoftwareX.

[68] Pronk S (2013). GROMACS 4.5: a high-throughput and highly parallel open source molecular simulation toolkit. Bioinformatics.

[69] Tribello GA, Bonomi M, Branduardi D, Camilloni C, Bussi G (2014). PLUMED 2: New feathers for an old bird. Comput. Phys. Commun..

[70] Ilott AJ, Palucha S, Hodgkinson P, Wilson MR (2013). Well-tempered metadynamics as a tool for characterizing multi-component, crystalline molecular machines. J. Phys. Chem. B.

[71] Fernández-Quintero, M. L., Pomarici, N. D., Seidler, C. A., Loeffler, J. R. & Liedl, K. R. T-cell receptor CDR3 loop confor mations in solution shift the relative V_H_-V_L_ domain distributions. *Front. Immunol*., 10.3389/fimmu.2020.01440 (2020). 10.3389/fimmu.2020.01440 PMC7360859 32733478

[72] Ramachandran GN, Ramakrishnan C, Sasisekharan V (1963). Stereochemistry of polypeptide chain configurations. J. Mol. Biol..

[73] Shao J, Tanner SW, Thompson N, Cheatham TE (2007). Clustering molecular dynamics trajectories: 1. Characterizing the performance of different clustering algorithms. J. Chem. Theory Comput..

[74] Case DA (2020). AMBER 2020.

[75] Miyamoto S, Kollman PA (1992). Settle: an analytical version of the SHAKE and RATTLE algorithm for rigid water models. J. Computat. Chem..

[76] Berendsen HJC, Postma JPM, van Gunsteren WF, DiNola A, Haak JR (1984). Molecular dynamics with coupling to an external bath. J. Chem. Phys..

[77] Adelman SA, Doll JD (1976). Generalized Langevin equation approach for atom/solid-surface scattering: general formulation for classical scattering off harmonic solids. J. Chem. Phys..

[78] Scherer MK (2015). PyEMMA 2: a software package for estimation, validation, and analysis of Markov models. J. Chem. Theory Comput..

[79] Chodera JD, Noé F (2014). Markov state models of biomolecular conformational dynamics. Curr. Opin. Struct. Biol..

[80] Likas A, Vlassis N, Verbeek JJ (2003). The global k-means clustering algorithm. Pattern Recognit..

[81] Röblitz S, Weber M (2013). Fuzzy spectral clustering by PCCA+: application to Markov state models and data classification. Adv. Data Anal. Classification.

[82] Bowman, G. R., Pande, V., Noé, F. (eds) *An Introduction to Markov State Models and Their Application to Long Timescale Molecular Simulation* (Springer, 2014).

[83] Miroshin RN (2016). Special solutions of the Chapman–Kolmogorov equation for multidimensional-state Markov processes with continuous time. Vestn. St. Petersburg Univ.: Math..

[84] Wu, H. & Noé, F. *Variational Approach for Learning Markov Processes from Time Series Data*10.1007/s00332-019-09567-y (2017).

[85] Dunbar J, Fuchs A, Shi J, Deane CM (2013). ABangle: characterising the V_H_–V_L_ orientation in antibodies. Protein Eng., Des. Selection.

[86] Bujotzek A (2016). VH-VL orientation prediction for antibody humanization candidate selection: acase study. mAbs.

[87] Stanfield RL, Zemla A, Wilson IA, Rupp B (2006). Antibody elbow angles are influenced by their light chain class. J. Mol. Biol..

