# Supplementary material for: Antibodies exhibit multiple paratope states influencing VH–VL domain orientations
Source: Commun Biol. 2020 Oct 20;3:589. doi: 10.1038/s42003-020-01319-z (PMC7576833; doi:10.1038/s42003-020-01319-z)
Supplement: Supplementary file 1 — Supplementary Information [file 42003_2020_1319_MOESM1_ESM.pdf]

Supplementary Table S1: Classification of kinetic minima in solution for the 2D7T antibody fragment into canonical structures for all CDR loops. The macrostates of the anti polyhydroxbutyrate antibody are color-coded as in Figure 1a/b, including the state probabilities in %. We observe transitions between different canonical clusters and dominant solution structures. Various canonical clusters are present within the same kinetic minimum in solution and thus might be combined. Below the average ABangle V_H-V_L interface angles (HL) for the individual macrostates are presented.

**2D7T**	Macrostate 1<br>6%	Macrostate 2<br>9%	Macrostate 3<br>36%	Macrostate 4<br>49%
**L1-11**				L1-11-1<br>L1-11-2
**L2-8**			L2-8-1<br>L2-8-4<br>L2-8-5	L2-8-2<br>L2-8-3
**L3-9**				L3-9-1<br>L3-9-2<br>L3-9-cis6-1<br>L3-9-cis7-1<br>L3-9-cis7-2<br>L3-9- cis7-3
**H1-13**				H1-13-4
**H2-10**	H2-10-3	H2-10-1<br>H2-10-8<br>H2-10-9	H2-10-6	H2-10-7<br>H2-10-5

**2D7T**	Macrostate 1<br>6%	Macrostate 2<br>9%	Macrostate 3<br>36%	Macrostate 4<br>49%
ABangle HL angle averages	-56.6°	-50.6°	-58.0°	-53.2°

Supplementary Table S2: Classification of kinetic minima in solution for the IL-18 human antibody fragment into canonical structures for all CDR loops. The macrostates of the IL-18 human antibody are color-coded as in Figure 3a/b, including the state probabilities in %. We observe transitions between different canonical clusters and dominant solution structures. Various canonical clusters are present within the same kinetic minimum in solution and thus might be combined. Below the average ABangle V_H-V_L interface angles (HL) for the individual macrostates are presented.

**2VXT**	Macrostate 1<br>3%	Macrostate 2<br>66%	Macrostate 3<br>31%
**L1-11**	L1-11-1<br>L1-11-2		
**L2-8**	L2-8-2<br>L2-8-3		L2-8-1<br>L2-8-4<br>L2-8-5

**L3-9**	L3-9-2<br>L3-9-cis6-1<br>L3-9-cis7-2<br>L3-9-cis7-3		L3-9-cis7-1
**H1-13**	H1-13-8		H1-13-1<br>H1-13-2<br>H1-13-4<br>H1-13-7
**H2-10**	H2-10-3<br>H2-10-8<br>H2-10-9	H2-10-7	H2-10-1<br>H2-10-6

**2VXT**	**Macrostate 1**<br>3%	**Macrostate 2**<br>66%	**Macrostate 3**<br>31%
ABangle HL angle averages	-70°	-68.8°	-69.45°

Supplementary Table S3: Classification of kinetic minima in solution for Fab-Hyb3 antibody fragment into canonical structures for all CDR loops. The macrostates of the Fab-Hyb3 are color-coded as in Figure 5a/b, including the state probabilities in %. We observe transitions between different canonical clusters and dominant solution structures. Various canonical clusters are present within the same kinetic minimum in

solution and thus might be combined. Below the average ABangle V_H-V_L interface angles (HL) for the individual macrostates are presented.

1W72	Macrostate 1 6%	Macrostate 2 8%	Macrostate 3 19%	Macrostate 4 67%
**L1-11**	L1-11-3			
**L2-8**	L2-8-3	L2-8-1<br>L2-8-2<br>L2-8-4<br>L2-8-5		
**L3-11**		L3-11-1		
**H1-13**			H1-13-1<br>H1-13-2<br>H1-13-7<br>H1-13-8<br>H1-13-4	
**H2-10**				H2-10-2<br>H2-10-4<br>H2-10-5<br>H2-10-7

1W72	Macrostate 1 6%	Macrostate 2 8%	Macrostate 3 19%	Macrostate 4 67%
ABangle HL angle averages	-62.6°	-63.8°	-62.6°	-61.9°

Supplementary Table S4: Classification of kinetic minima in solution for the naïve D44.1 antibody fragment into canonical structures for all CDR loops. The macrostates of the naïve D44.1 antibody is color-coded as in Figure 8a/b, including the state probabilities in %. We observe transitions between different canonical clusters and dominant solution structures. Various canonical clusters are present within the same kinetic minimum in

solution and thus might be combined. Below the average Below the average ABangle V_H-V_L interface angles (HL) for the individual macrostates are presented.

1MLC	Macrostate 2 18%	Macrostate 3 35%	Macrostate 4 7%	Macrostate 5 9%	Macrostate 6 23%
**L1-11**				L1-11-1<br>L1-11-2	
**L2-8**	L2-8-5	L2-8-1<br>L2-8-2<br>L2-8-4		L2-8-3	
**L3-9**		L3-9-cis7-3		L3-9-2	L3-9-cis6-1<br>L3-9-cis7-1<br>L3-9-cis7-2
**H1-13**	H1-13-2<br>H1-13-7<br>H1-13-8	H1-13-1			H1-13-4
**H2-10**		H2-10-9	H2-10-1<br>H2-10-6	H2-10-7	

1MLC	Macrostate 1 8%	Macrostate 2 18%	Macrostate 3 35%	Macrostate 4 7%	Macrostate 5 9%	Macrostate 6 23%
ABangle HL angle averages	-56.4°	-64.3°	-63.5°	-61.0°	-64.3°	-62.3°

Supplementary Table S5: Classification of kinetic minima in solution for the matured F10.6.6 antibody fragment into canonical structures for all CDR loops. The macrostates of the matured F10.6.6 antibody is color-coded as in Figure 9a/b, including the state probabilities in %. We observe transitions between different canonical clusters and dominant solution structures. Various canonical clusters are present within the same

kinetic minimum in solution and thus might be combined. Below the average ABangle V_H-V_L interface angles (HL) for the individual macrostates are presented.

**1P2C**	**Macrostate 1** 12%	**Macrostate 2** 44%	**Macrostate 3** 44%
**L1-11**			L1-11-1<br>L1-11-2
**L2-8**		L2-8-1<br>L2-8-2<br>L2-8-3<br>L2-8-4<br>L2-8-5	
**L3-9**		L3-9-2<br>L3-9-cis6-1<br>L3-9-cis7-1<br>L3-9-cis7-2	L3-9-cis7-3
**H1-13**	H1-13-1<br>H1-13-2<br>H1-13-7	H1-13-8	H1-13-4
**H2-10**	H2-10-1<br>H2-10-6	H2-10-8<br>H2-10-9	H2-10-3

**1P2C**	**Macrostate 1** 12%	**Macrostate 2** 44%	**Macrostate 3** 44%
ABangle HL angle averages	-61.8°	-61.1°	-59.6°

Supplementary Table S6: Classification of kinetic minima in solution for the SPE7 antibody fragment into canonical structures for all CDR loops. The macrostates of the SPE7 antibody is color-coded as in Figure 7a/b, including the state probabilities in %. We observe transitions between different canonical clusters and dominant solution structures. Various canonical clusters are present within the same kinetic minimum in solution and thus might be combined. Below the average ABangle V_H-V_L interface angles for the individual macrostates are presented.

1OAQ	Macrostate 1 25%	Macrostate 2 32%	Macrostate 3 43%
**L1-11**		L1-14-1	
**L2-8**		L2-8-1<br>L2-8-2<br>L2-8-3<br>L2-8-4<br>L2-8-5	
**L3-11**		L3-9-1	L3-9-2<br>L3-9-cis6-1<br>L3-9-cis7-1<br>L3-9-cis7-2<br>L3-9-cis7-3
**H1-13**	H1-13-1<br>H1-13-7	H1-13-2<br>H1-13-4<br>H1-13-8	
**H2-10**	H2-10-2<br>H2-10-4<br>H2-10-7	H2-10-5<br>H2-10-9	H2-10-1<br>H2-10-3<br>H2-10-6<br>H2-10-8

1OAQ	Macrostate 1 25%	Macrostate 2 32%	Macrostate 3 43%
ABangle HL angle averages	-63.5°	-60.0°	-59.6°

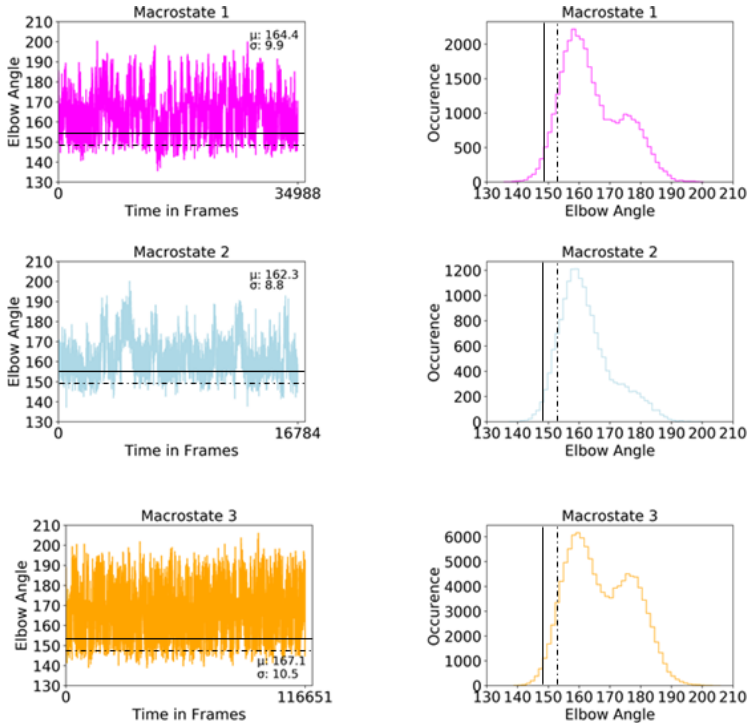

Supplementary Figure S1: Elbow-angle distributions of the different CDR loop macrostates to investigate the role of different binding interface state on the elbow-angle. The color-coding of the distributions corresponds to the macrostate ensembles and the VH-VL interface distributions, illustrated in Figure 3b. We clearly observe differences in the elbow-angle distributions, upon changes in the binding interface. The horizontal and vertical lines represent the available X-ray structures with the PDB accession code 2VXT. The dashed line in this case represents the unbound structure with the PDB accession code 2VXU, showing that we can sample the 5° difference in the elbow-angle, due to crystal packing effects.

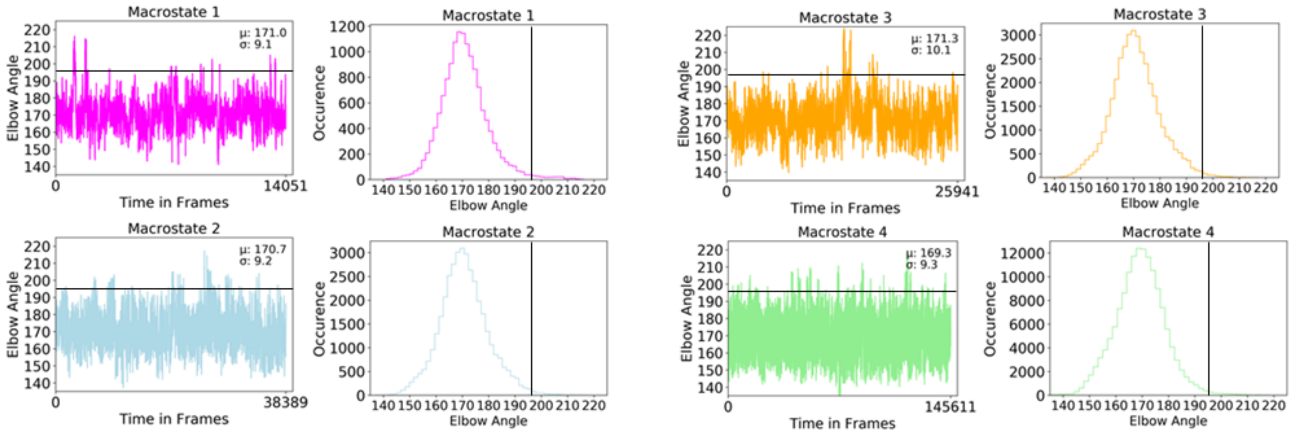

Supplementary Figure S2: *Elbow-angle distributions of the different CDR loop macrostates to investigate the role of different binding interface state on the elbow-angle. The color-coding of the distributions corresponds to the macrostate ensembles and the VH-VL interface distributions, illustrated in Figure 5b. We observe nearly no differences in elbow-angle distributions upon differences in the binding interface. The horizontal and vertical lines represent the available X-ray structures with the PDB accession code 1W72.*

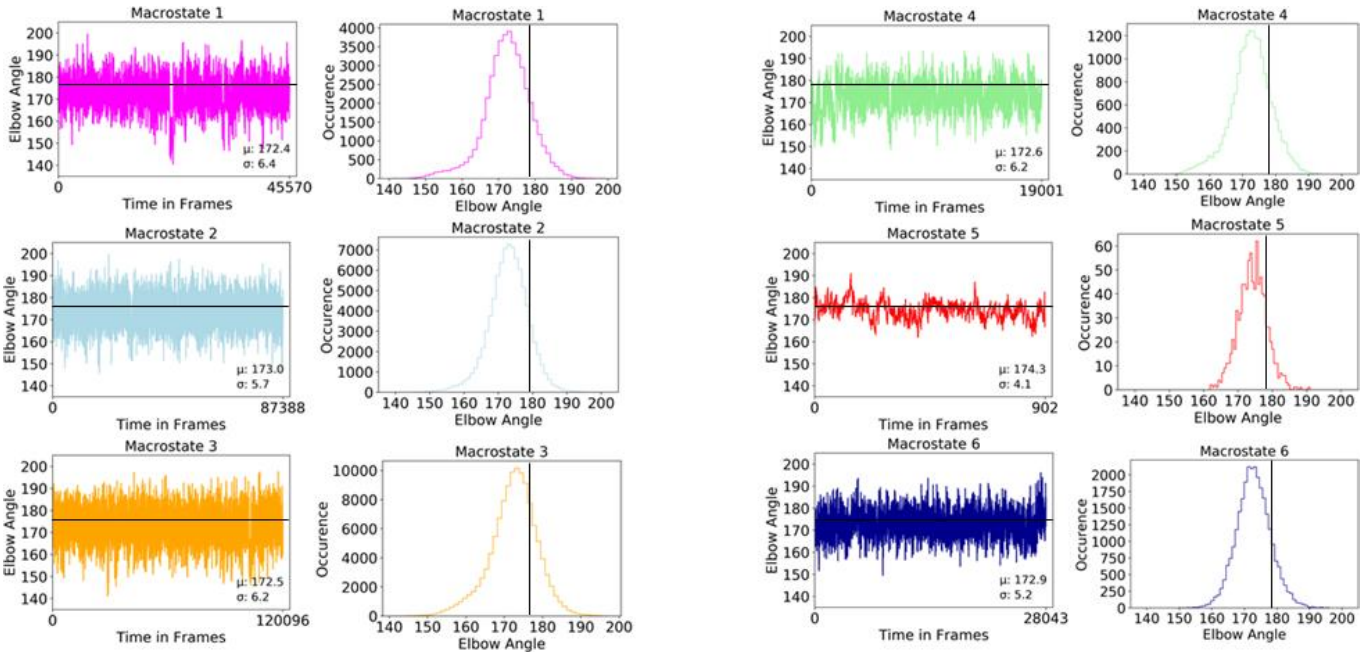

Supplementary Figure S3: Elbow-angle distributions of the different CDR loop macrostates to investigate the role of different binding interface state on the elbow-angle. The color-coding of the distributions corresponds to the macrostate ensembles and the VH-VL interface distributions, illustrated in Figure 8b. We observe small shifts in elbow-angle distributions upon differences in the binding interface. The horizontal and vertical lines represent the available X-ray structures with the PDB accession code 1MLC.

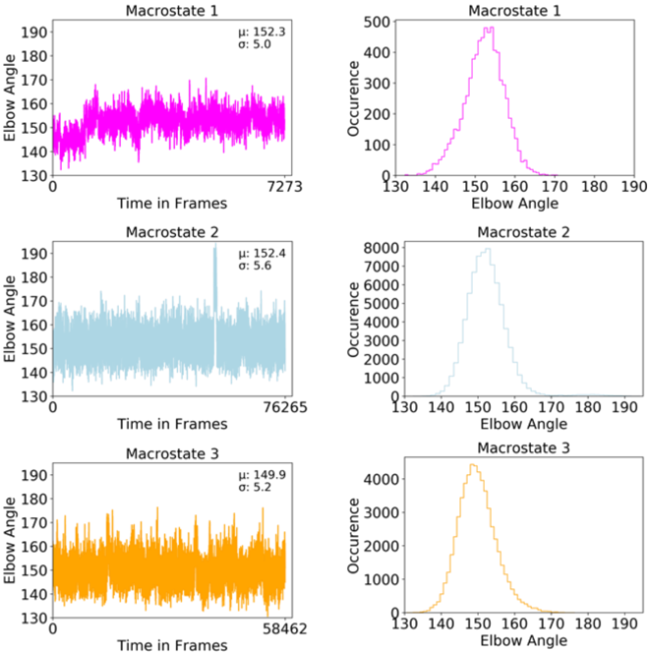

Supplementary Figure S4: Elbow-angle distributions of the different CDR loop macrostates to investigate the role of different binding interface state on the elbow-angle. The color-coding of the distributions corresponds to the macrostate ensembles and the VH-VL interface distributions, illustrated in Figure 9b. We observe small shifts in elbow-angle distributions upon differences in the binding interface. The horizontal and vertical lines represent the available X-ray structures with the PDB accession code 1P2C.

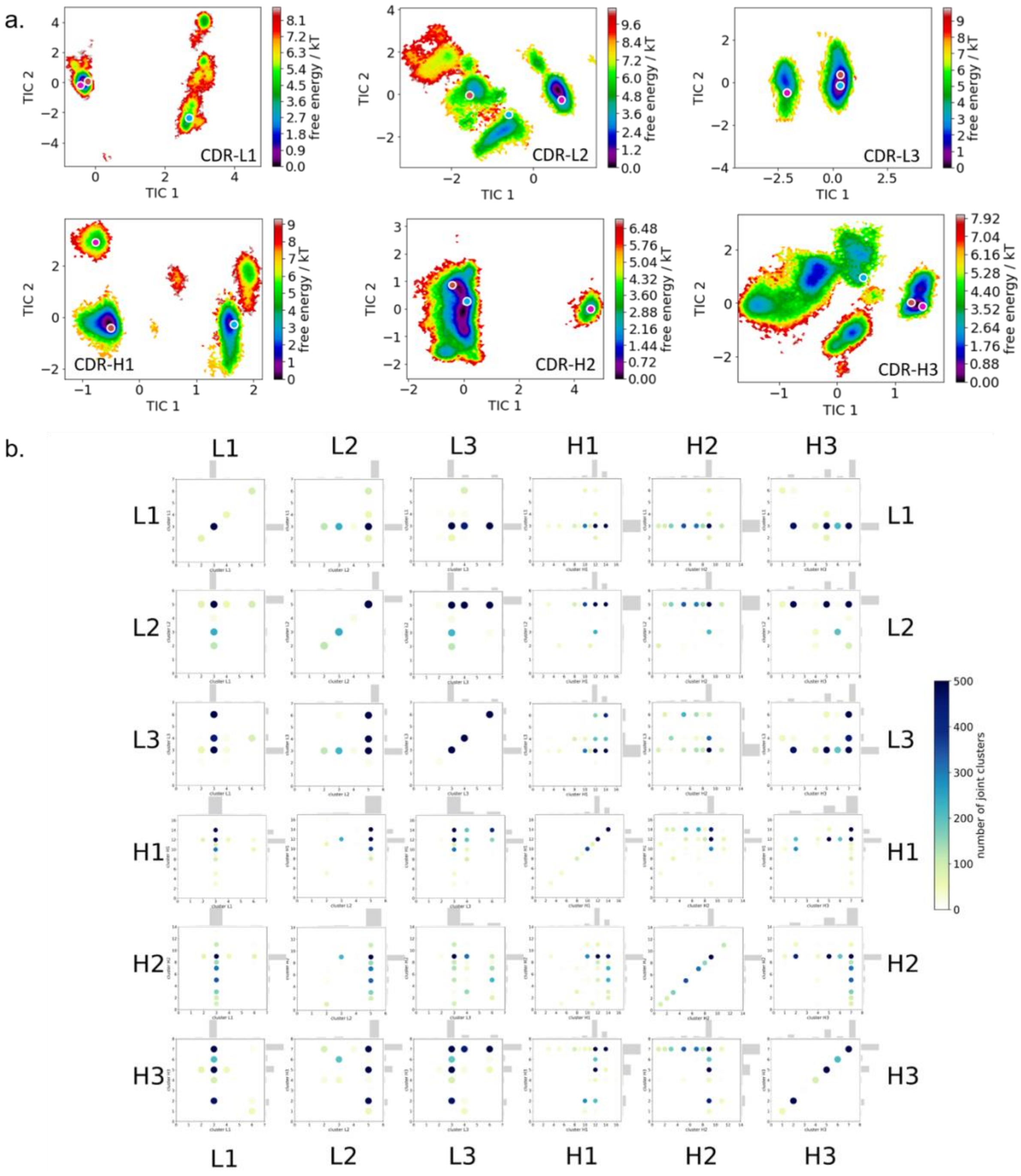

Supplementary Figure S5: Free energy surfaces of all individual CDR loops of the SPE7 antibody including the projections of macrostates of Figure 7b). b) Visualization of simultaneous occurrence of CDR loop conformations for all CDR loops.

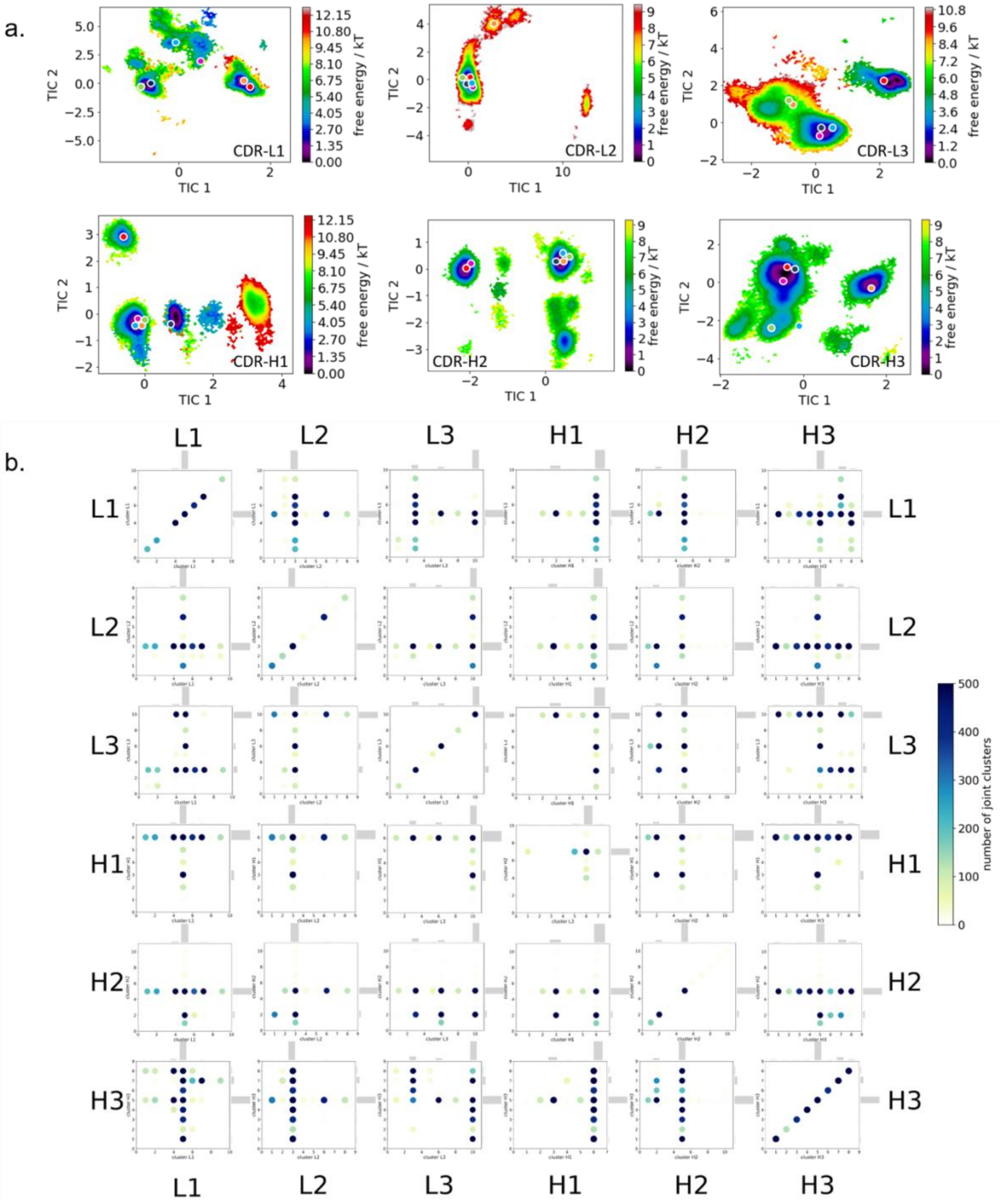

Supplementary Figure S6: a) Free energy surfaces of all individual CDR loops of the naïve D44.1 antibody including the projections of macrostates of Figure 8b). b) Visualization of simultaneous occurrence of CDR loop conformations for all CDR loops.

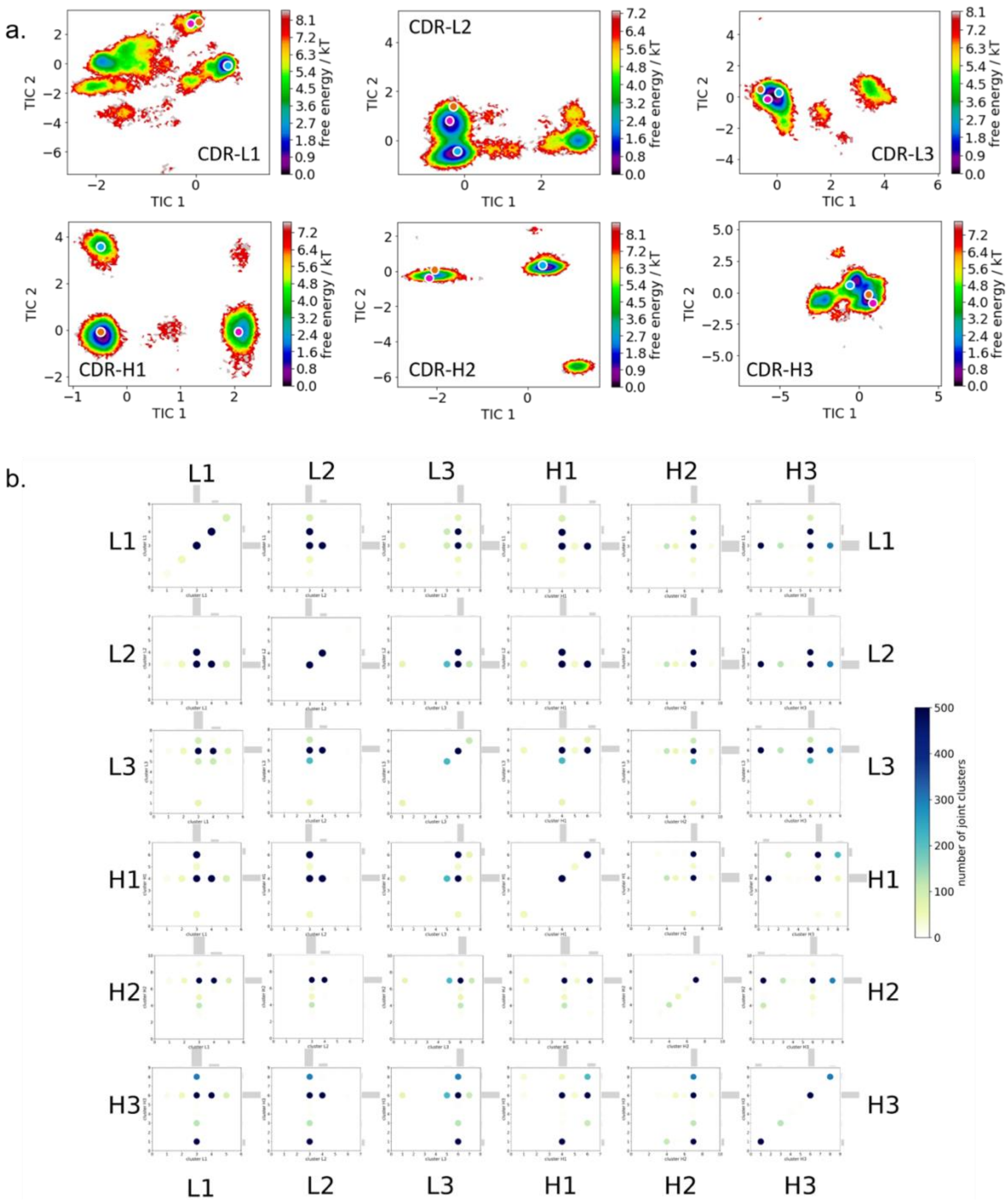

Supplementary Figure S7: a) Free energy surfaces of all individual CDR loops of the matured F10.6.6 antibody including the projections of macrostates of Figure 9b). b) Visualization of simultaneous occurrence of CDR loop conformations for all CDR loops.

.

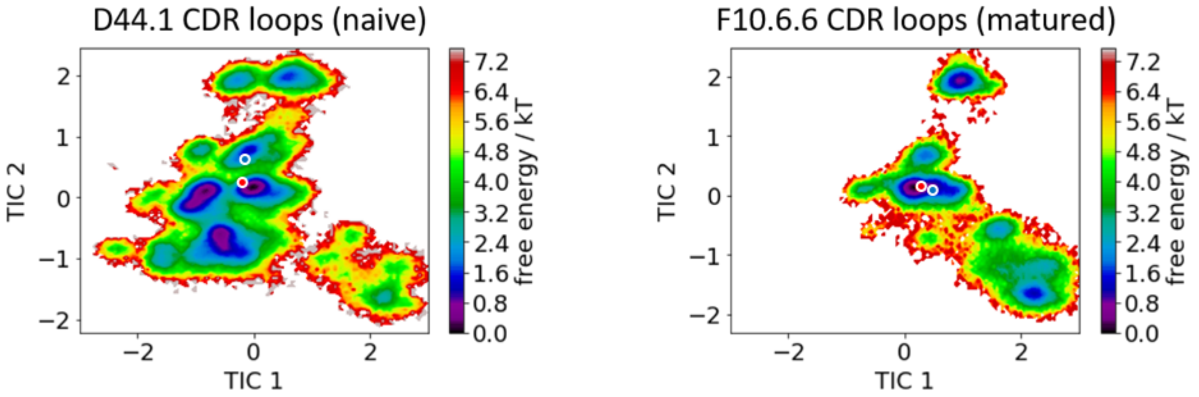

Supplementary Figure S8: The free energy surface of the naive D44.1 antibody (left) reveals a substantial broader CDR loop conformational space compared to the matured F10.6.6. antibody. In red the starting crystal structures with the PDB accession codes 1MLC and 1P2C, respectively, are shown. Both crystal structures were crystallized with the presence of the antigen. The available crystal structures, without the antigen present, are shown in blue.

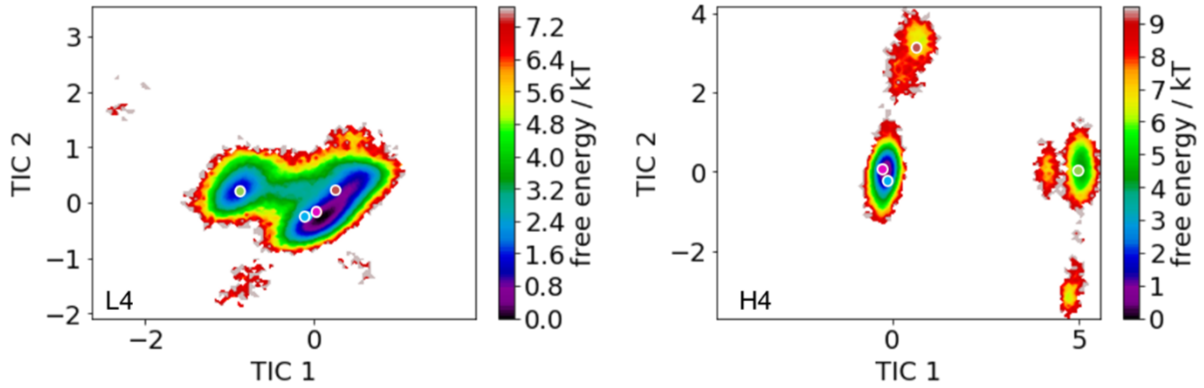

Supplementary Figure S9: Free energy surfaces of the L4 (left) and H4 (right) loops with the respective macrostate representatives of the SPE7 antibody. Also, for the L4/H4 loops we observe conformational changes upon rearrangements in the CDR binding loops. Additionally, we observe different macrostates (2 on the left and three on the right side) in solution, indicating that also these loops are flexible and can adopt various conformations in solution.

Supplementary Table S7: Summary table of the elbow-angle averages for each macrostate of each antigen-binding fragment studied.

PDB	Macrostate 1	Macrostate 2	Macrostate 3	Macrostate 4	Macrostate 5	Macrostate 6
1W72	158.69°	159.83°	160.83°	157.91°		
2VXT	159.48°	155.5°	162.58°			
1MLC	162.54°	164.30°	162.58°	164.10°	161.50°	161.27°
1P2C	132,77°	134,29°	131,25°			

Supplementary Table S8: Summary table of the ABangle averages for each macrostate of each antigen-binding fragment studied.

PDB	Macrostate 1	Macrostate 2	Macrostate 3	Macrostate 4	Macrostate 5	Macrostate 6
2D7T	-56.6°	-50.6°	-58.0°	-53.2°		
2VXT	-70.0°	-68.8°	-69.45°			
1W72	-62.6°	-63.8°	-62.6°	-61.9°		
1OAQ	-63.5°	-60.0°	-59.6°			
1MLC	-56.4°	-64.3°	-63.5°	-61.0°	-64.3°	-62.3°
1P2C	-61.8°	-61.1°	-59.6°			

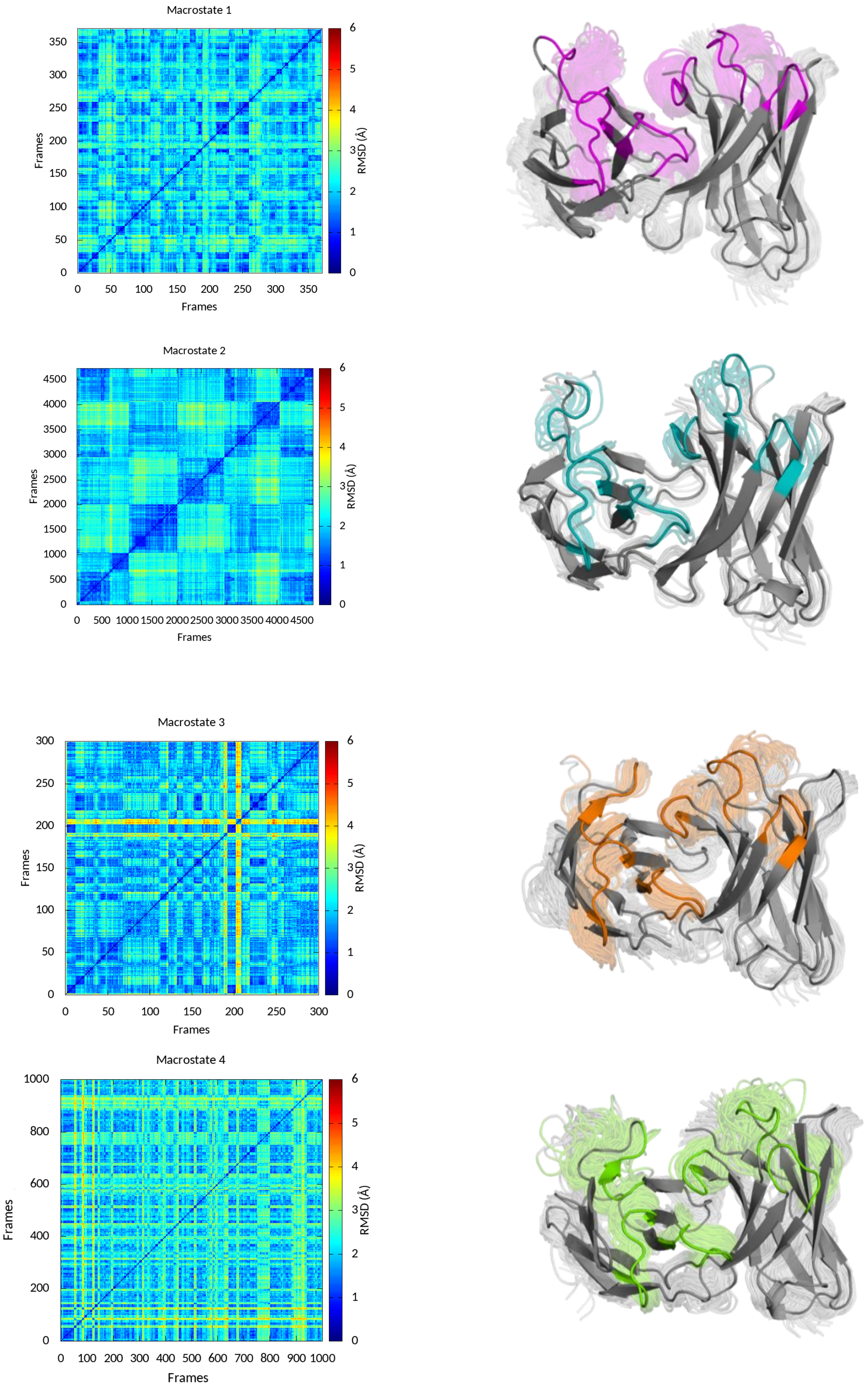

Supplementary Figure S10: 2D-rmsd plots of the anti polyhydroxbutyrat antibody for the individual macrostates including the respective macrostate ensembles depicted on the right.

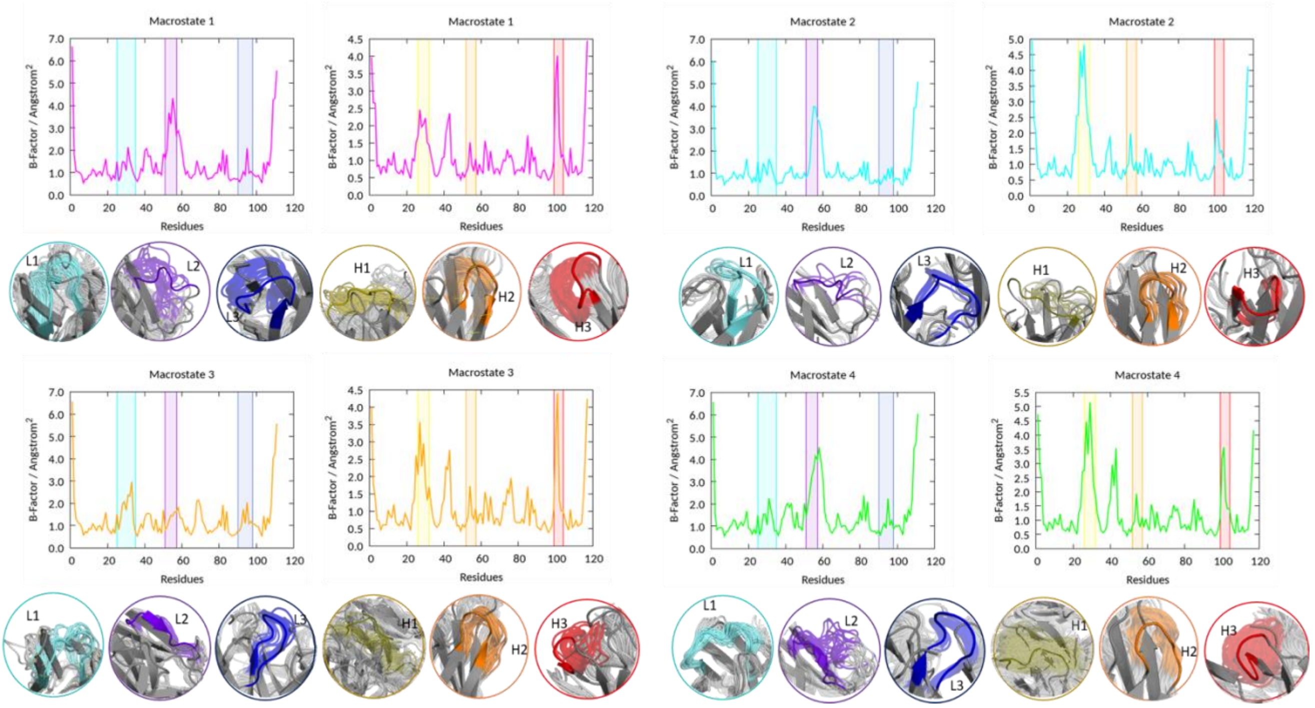

Supplementary Figure S11: B-factors for the individual macrostates to characterize the conformational diversity within and between the different macrostates for the anti polyhydroxbutyrate antibody. The vertical boxes highlight the respective CDR loops of both the heavy and the light chain and the color coding corresponds to the loop ensemble shown below.

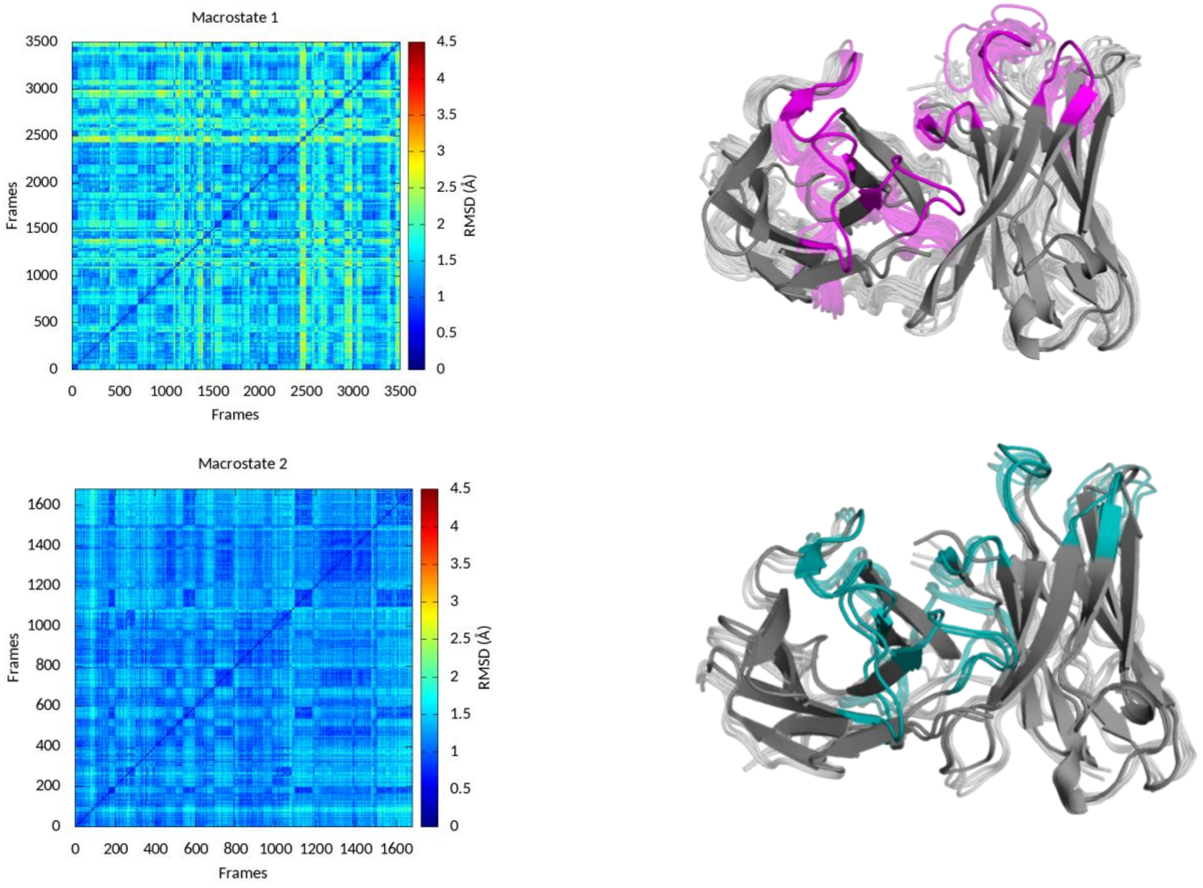

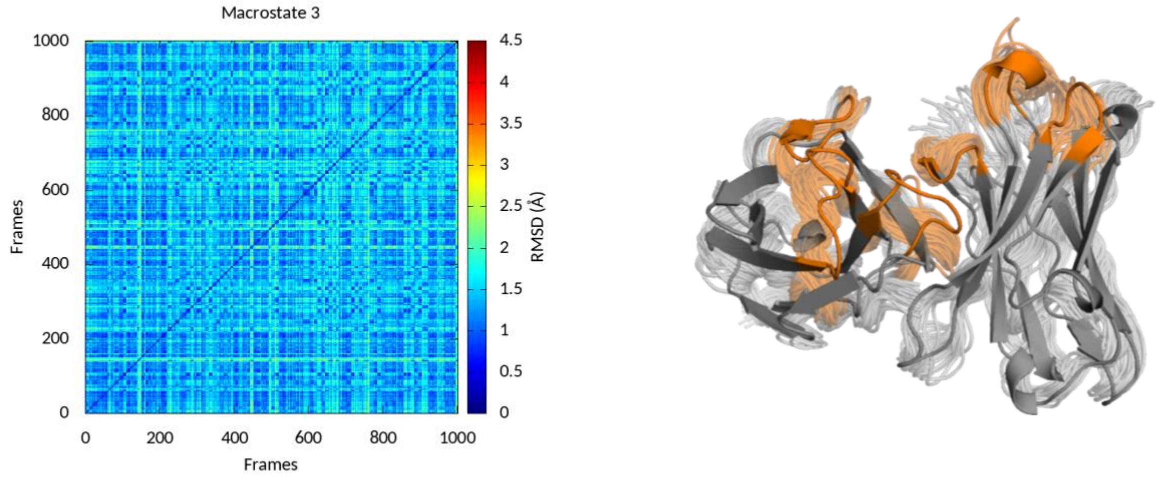

Supplementary Figure S12: 2D-rmsd plots of the individual macrostates to characterize the conformational diversity within and between the different macrostates for the IL-18 binding antibody.

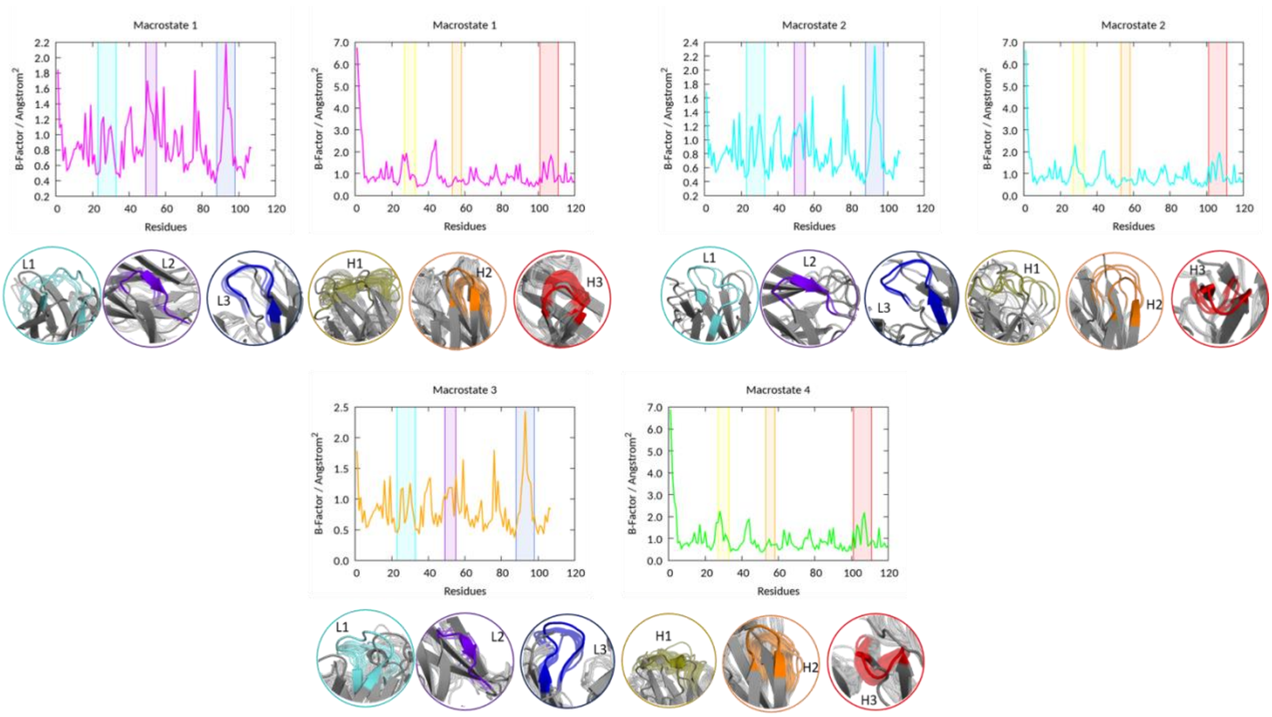

Supplementary Figure S13: B-factors for the individual macrostates to characterize the conformational diversity within and between the different macrostates for the IL-18 antibody. The vertical boxes highlight the respective CDR loops of both the heavy and the light chain and the color coding corresponds to the loop ensemble shown below.

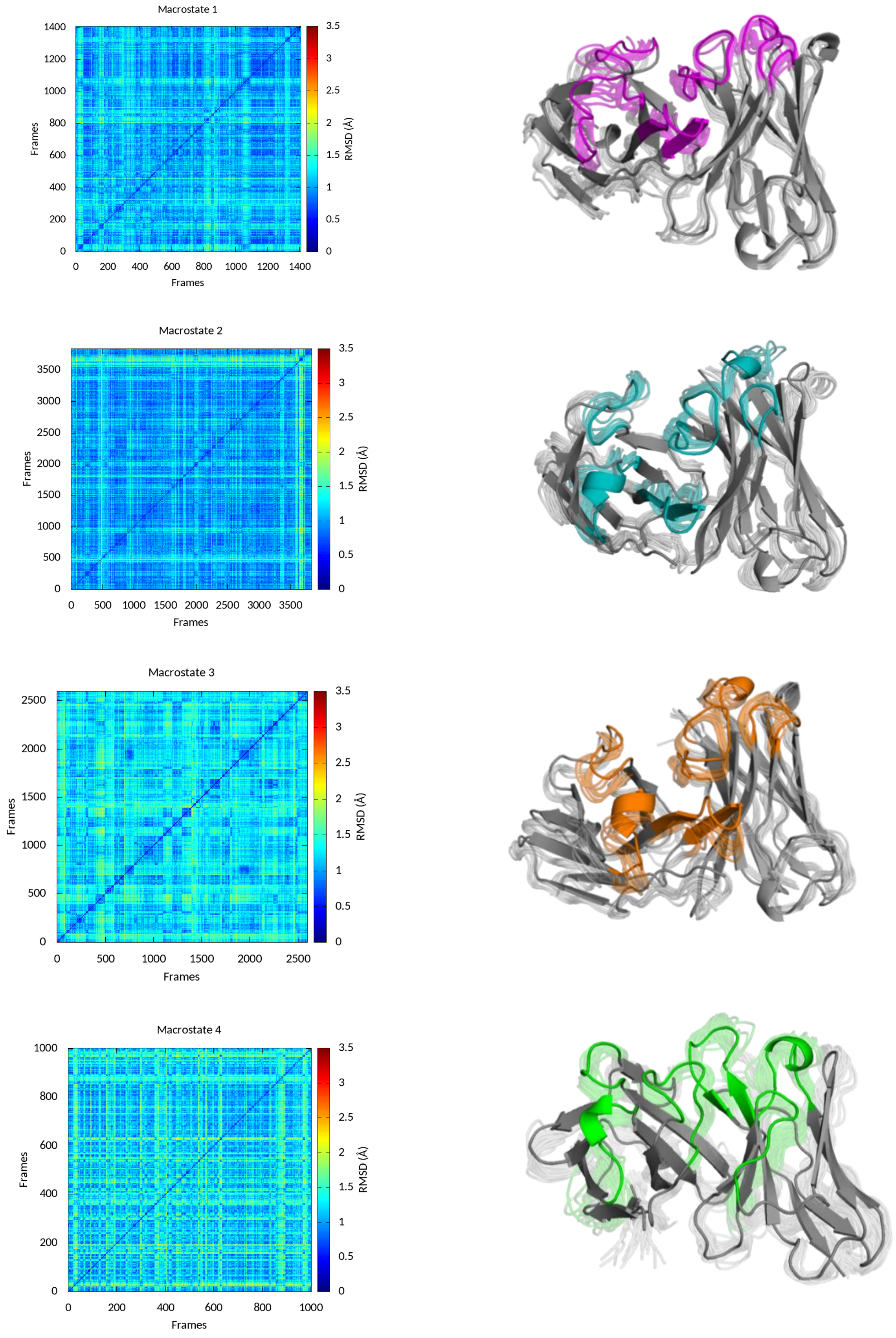

Supplementary Figure S14: 2D-rmsd plots of the individual macrostates to characterize the conformational diversity within and between the different macrostates for the Fab-Hyb3 binding antibody.

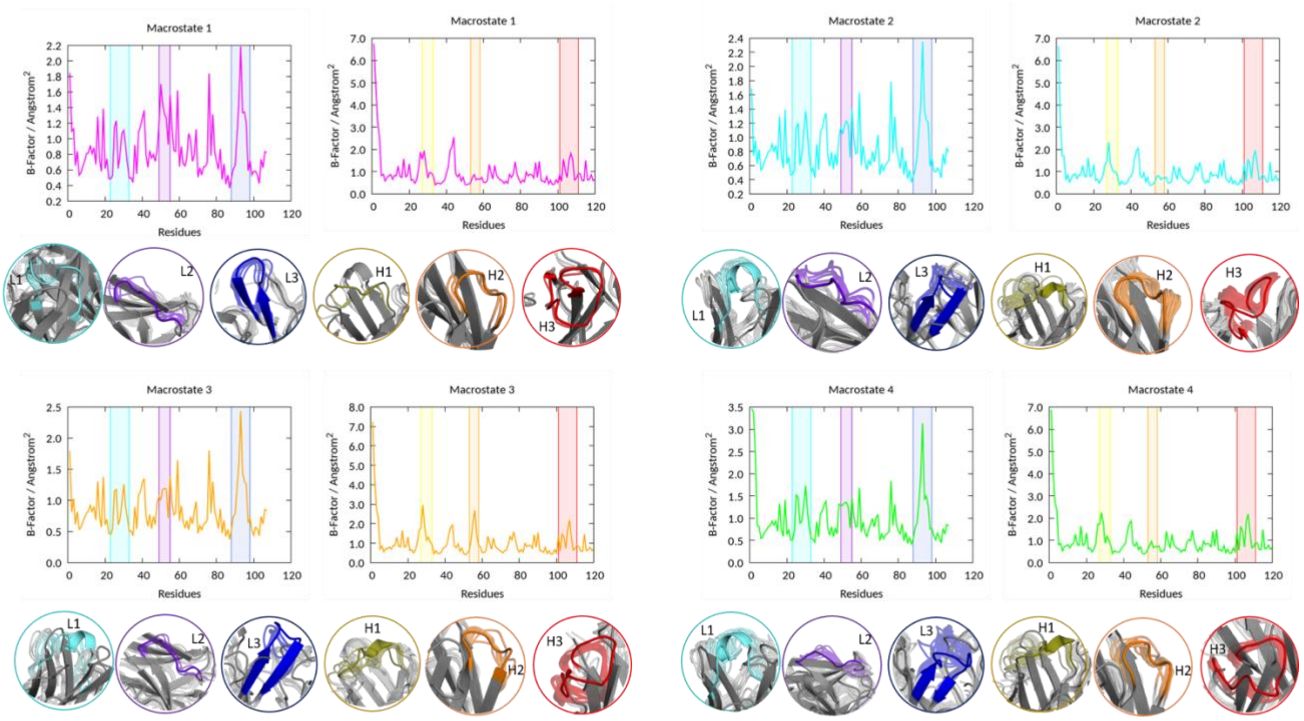

Supplementary Figure S15: B-factors for the individual macrostates to characterize the conformational diversity within and between the different macrostates for the Fab-Hyb3 antibody. The vertical boxes highlight the respective CDR loops of both the heavy and the light chain and the color coding corresponds to the loop ensemble shown below.

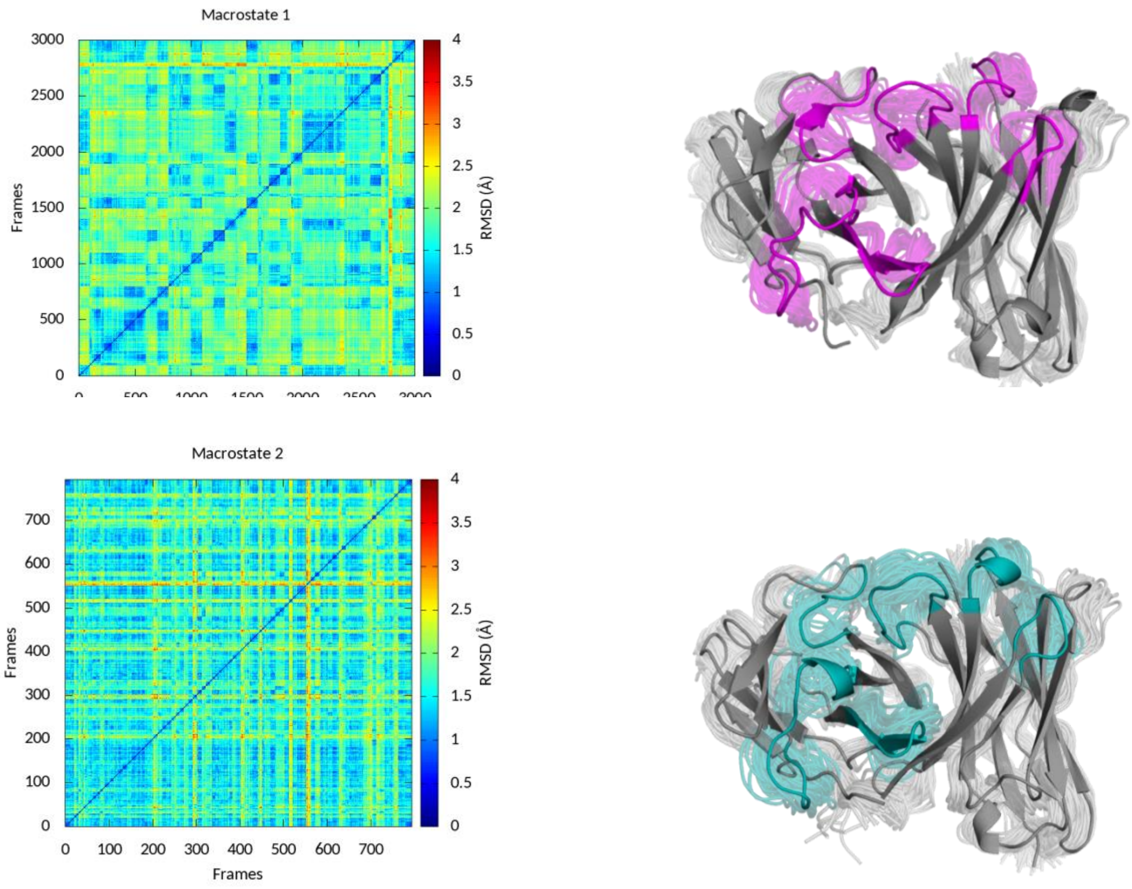

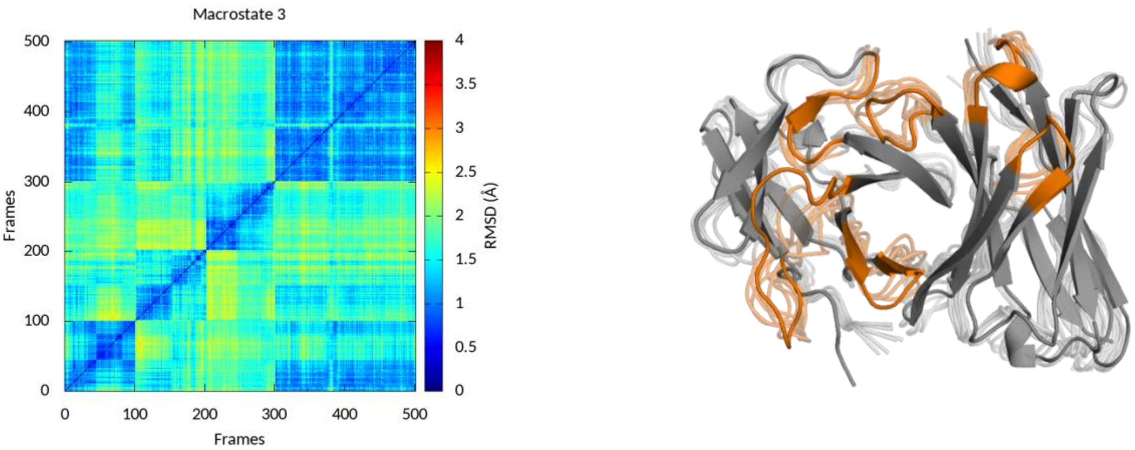

Supplementary Figure S16: 2D-rmsd plots of the individual macrostates to characterize the conformational diversity within and between the different macrostates for the SPE7 binding antibody.

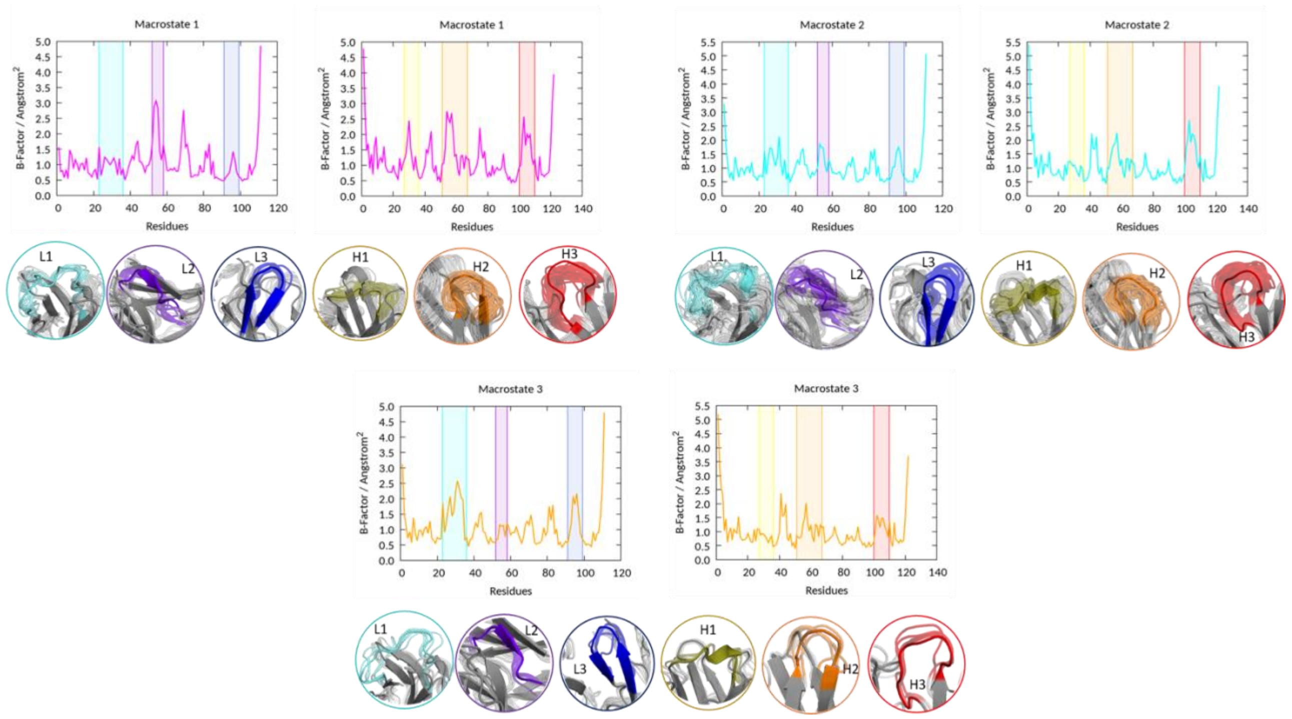

Supplementary Figure S17: B-factors for the individual macrostates to characterize the conformational diversity within and between the different macrostates for the SPE7 antibody. The vertical boxes highlight the respective CDR loops of both the heavy and the light chain and the color coding corresponds to the loop ensemble shown below.

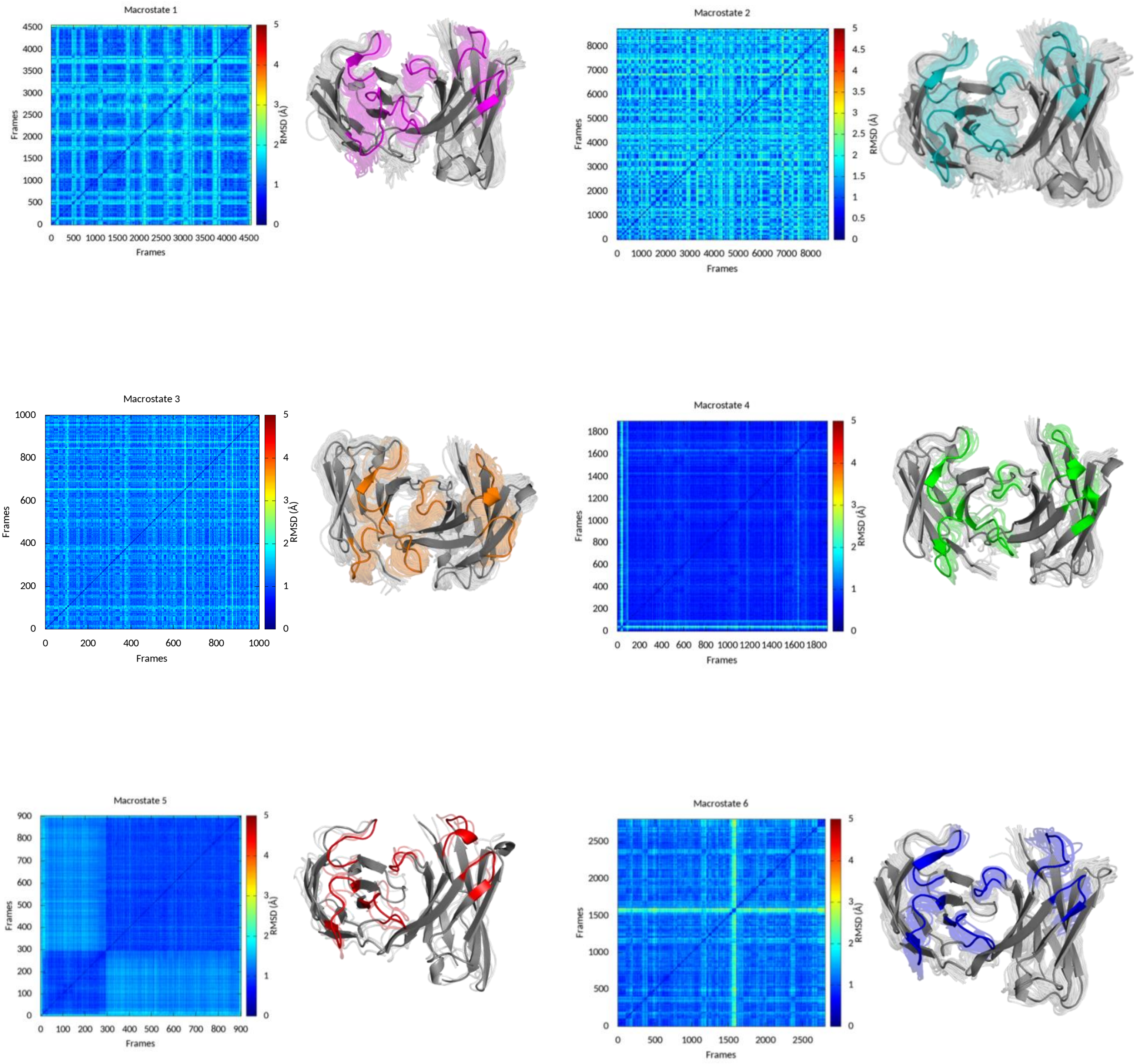

Supplementary Figure S18: 2D-rmsd plots of the individual macrostates to characterize the conformational diversity within and between the different macrostates for the D44.1 binding antibody.

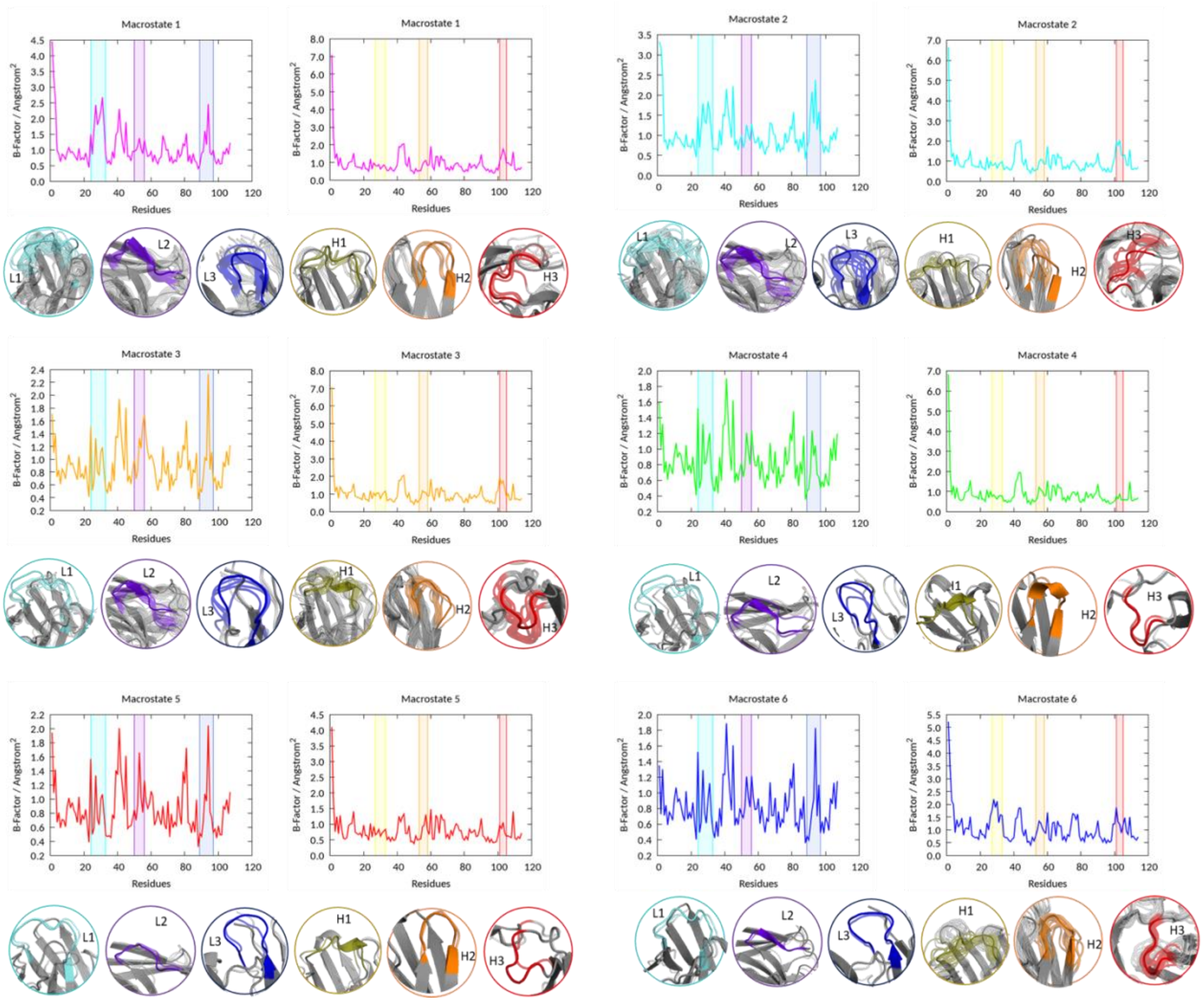

Supplementary Figure S19: B-factors for the individual macrostates to characterize the conformational diversity within and between the different macrostates for the D44.1 antibody. The vertical boxes highlight the respective CDR loops of both the heavy and the light chain and the color coding corresponds to the loop ensemble shown below.

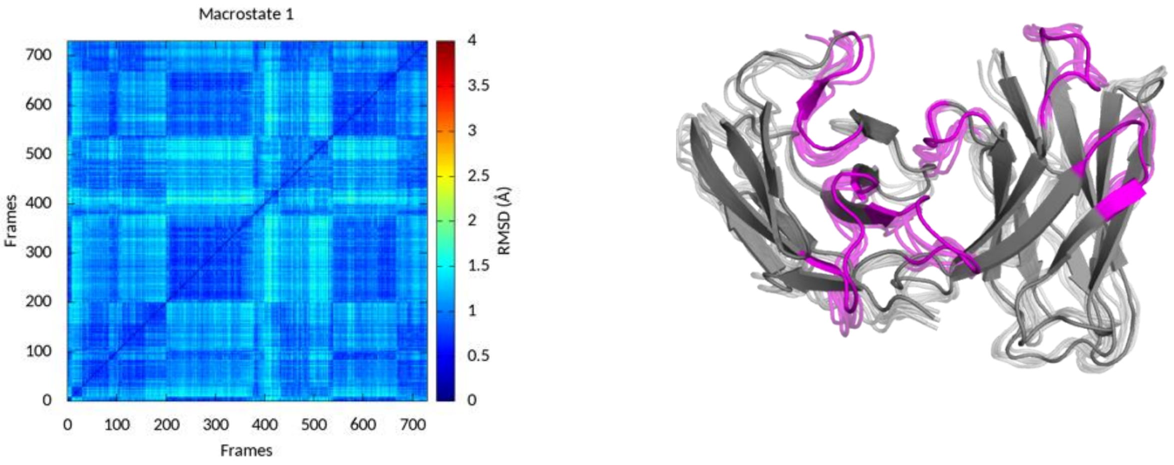

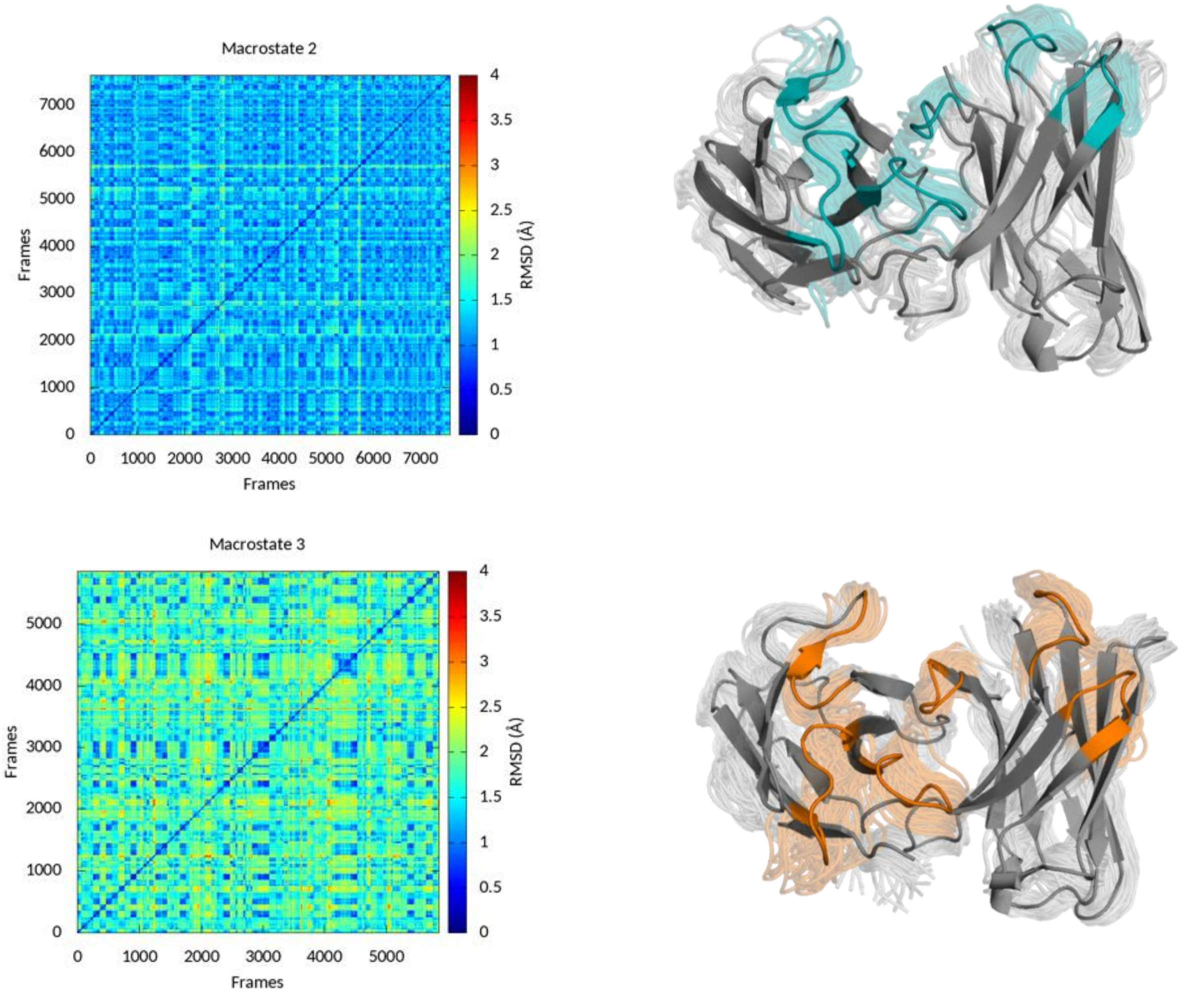

Supplementary Figure S20: 2D-rmsd plots of the individual macrostates to characterize the conformational diversity within and between the different macrostates for the F10.6.6 binding antibody.

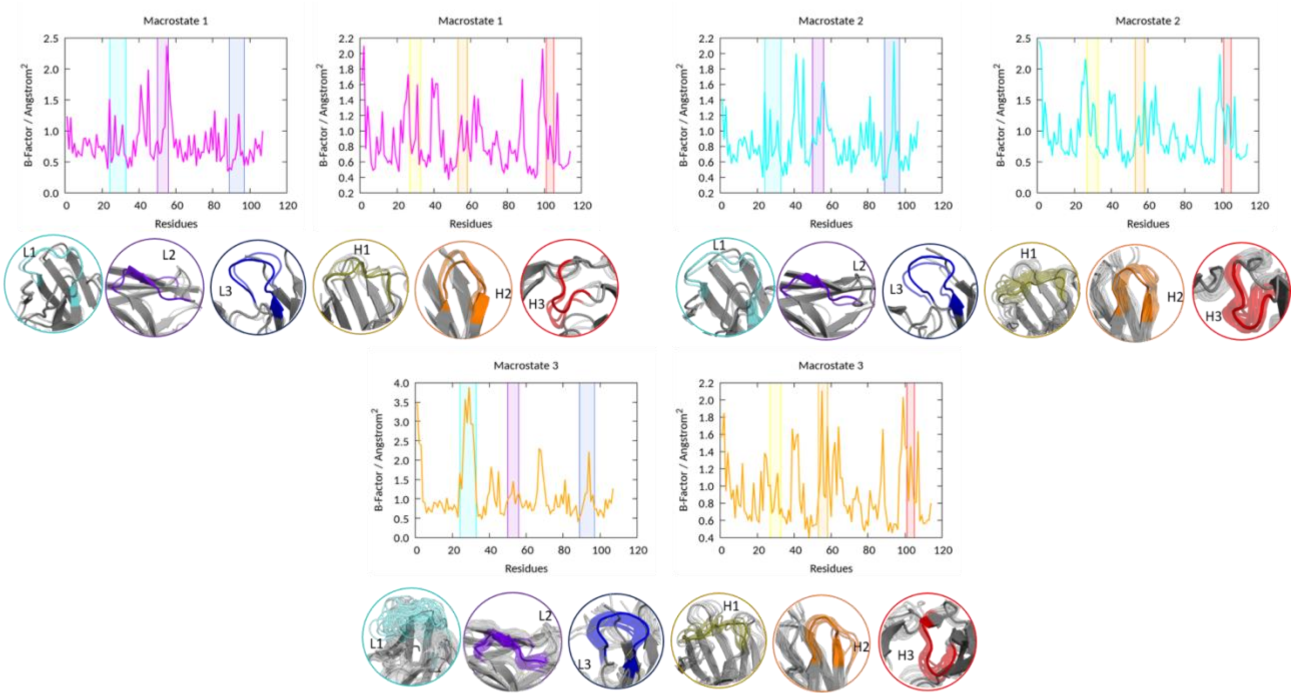

Supplementary Figure S21: B-factors for the individual macrostates to characterize the conformational diversity within and between the different macrostates for the F10.6.6 antibody. The vertical boxes highlight the respective CDR loops of both the heavy and the light chain and the color coding corresponds to the loop ensemble shown below.

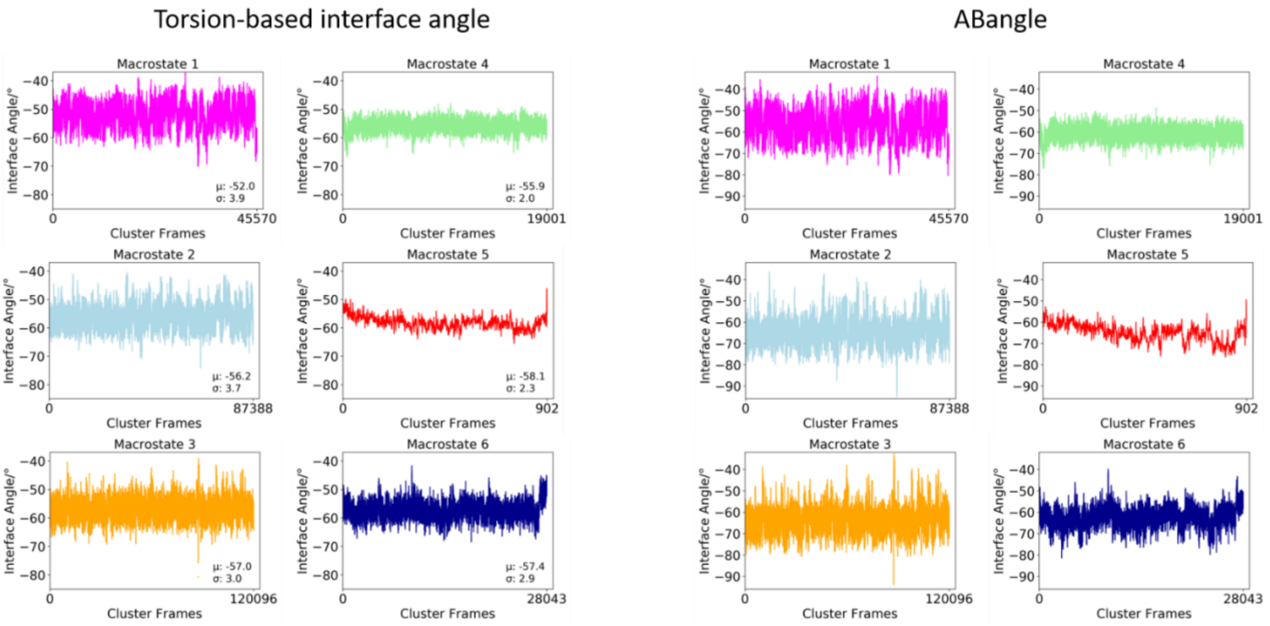

Supplementary Figure S22: Comparison between the torsion-based interface angle and the ABangle HL angle distributions for the individual macrostates of the D44.1 antibody. We chose this system as conformational rearrangements of the CDR loops have clearly shown to shift the relative interdomain distributions. This shift in the relative interdomain orientations can be seen independent of the method used.
